# Highly efficient Lewis acid catalytic activity of the tritylium ion at the node of a tensile organic framework†

**DOI:** 10.1039/d1sc02594e

**Published:** 2021-06-11

**Authors:** Shuai Zhao, Juhui Zhang, Yongchang Zhai, Xiaoqin Zou, Shaolei Wang, Zheng Bian, Fengchao Cui, Guangshan Zhu

**Affiliations:** Key Laboratory of Polyoxometalate and Reticular Material Chemistry of the Ministry of Education, Faculty of Chemistry, Northeast Normal University Changchun 130024 China bianz070@nenu.edu.cn cuifc705@nenu.edu.cn zhugs100@nenu.edu.cn

## Abstract

Tritylium salts have been used as Lewis acid catalysts in organic synthesis for a long time. In this work, we found that the Lewis acid catalytic activity of tritylium ions at the node of a tensile framework is significantly improved compared to that of the free tritylium salts. The tritylium-based framework, PAF-201 (PAF, porous aromatic framework), was prepared by acidification of a semi-rigid triphenylcarbinol-based parent framework, PAF-200. When PAF-200 was alternately exposed to HCl and NH_3_ gas, a fast allochroic cycle was observed due to repeated formation of tritylium species. Interestingly, the pseudo-first-order reaction rate of a Povarov model reaction catalyzed by PAF-201 as a Lewis acid was ∼3.7 times and ∼4.7 times as those of tritylium tetrafluoroborate and tri(4-biphenyl)carbonium tetrafluoroborate, respectively. Theoretical calculations revealed that the tritylium ion at the node of PAF-201 has a quasi-planar structure. The transformation of triphenylcarbinol in PAF-200 to tritylium in PAF-201 can make the framework taut, and the rebounding force toward the tetrahedral structure is stored. This is favorable for tritylium to activate the imine substrate along with a deformation of the quasi-plane to tetrahedron. PAF-201 could be easily recycled at least three times without evident loss of catalytic activity. This work presents the catalytic activity of the tritylium ion under stress.

## Abstract 2

In this work, a tensile tritylium-based organic framework, PAF-201 was prepared. The Lewis acid catalytic ability of PAF-201 was significantly higher than that of tritylium tetrafluoroborate. This work presented the catalytic activity of the tritylium ion under stress.

## Graphical abstract

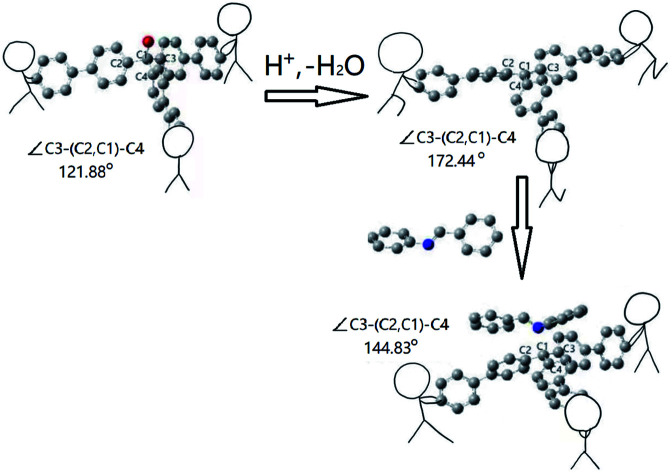

## Introduction

Porous organic polymers (POPs) have been developed rapidly owing to their significant advantages, such as large specific surface area and high stability.^1–3^ In particular, introducing an ionic structure into the polymer backbone endows POPs with new functionalities that may expand their applications.^4–7^ These backbones with negative or positive charges have been applied to different aspects, such as transport, absorption, separation, or catalysis.^4–7^ Notably, in terms of catalysis, most ionic frameworks have been used to carry active ionic counterparts or metal nanoparticles, and have seldom been used as catalytic sites themselves. In addition, research on porous ionic polymers has mainly focused on boron-,^8^ nitrogen-,^9^ oxygen-^10^ or phosphorus-centered ions,^11^ but seldom on carbon-centered ions by now.^12^

As we know, tritylium salts are widely used in organic reactions, especially as Lewis acid catalysts and dehydrogenative reagents.^13^ However, their use is limited by their high price and difficult recovery. Notably, porous aromatic frameworks (PAFs) have high-porosity structures, chemical affinities that can be tuned through the choice of node and linker, and extraordinary hydrothermal stability and chemical compositions.^14^ In this work, we plan to introduce tritylium units into the PAF framework so as to obtain an easily recycled material with the properties of tritylium salts. Considering that tritylium salts are generally prepared by acidification of triphenylcarbinol, it is a simple idea to use a triphenylcarbinol-based framework as the precursor for a tritylium-based one. Notably, the tritylium ion is the most stable carbocation with a propeller-like conformation, and partial charge of the central carbenium is dispersed on three phenyl rings. There will be a big deformation from triphenylcarbinol to tritylium. Thus, it is necessary for the parent framework to withdraw the tension due to this deformation. Some triphenylcarbinol-based porous materials have been reported,^15–16^ but the corresponding tritylium-based frameworks as Lewis acids have not been introduced so far. Herein, the tritylium-based framework was first prepared. To our surprise, it was found that the tritylium ions at the node of the tensile framework exhibited better Lewis acid catalytic ability than the free ones.

## Results and discussion

### Synthesis and characterization of PAF-200 and PAF-201

**Scheme 1:**
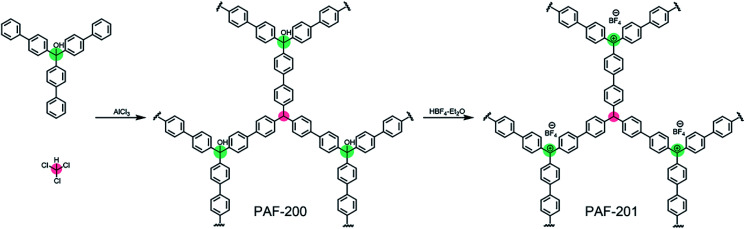
The synthesis of PAF-200 and PAF-201.

**Fig. 1:**
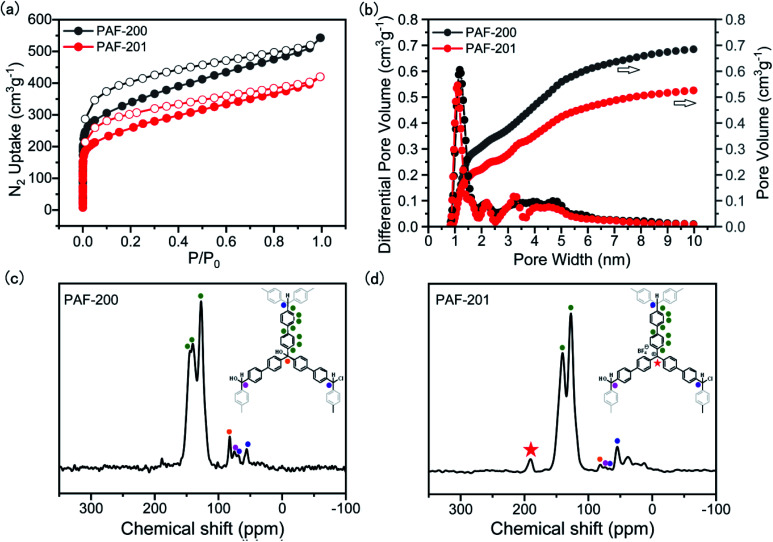
N_2_ adsorption and desorption isotherms at 77 K (a), QSDFT-derived pore size distributions (b) and solid-state ^13^C CP/MAS NMR spectra of PAF-200 (c) and PAF-201 (d).

First, we tried to synthesize a porous framework using commercially available triphenylcarbinol as the substrate by AlCl_3_-activated Friedel–Crafts reaction in CHCl_3_. The resultant material exhibited very low porosity (Fig. S1†), possibly due to the small size of the substrate. Furthermore, bulky tri(4-biphenyl)-carbinol (TBOH) was chosen as the substrate using a similar method to afford the porous material PAF-200 (Scheme 1). Subsequently, the tritylium-based porous framework PAF-201 was obtained *via* HBF_4_ treatment of PAF-200. Fig. 1a and b presents the nitrogen adsorption–desorption isotherms of the two PAFs at 77 K, and the details of the porous properties are summarized in Table S1.† Rapid adsorption occurred at low relative pressure, characteristic for microporous materials. The Brunner–Emmet–Teller (BET) surface areas of PAF-200 and PAF-201 were 1234 m^2^ g^−1^ and 917 m^2^ g^−1^, respectively. Both PAFs possessed dominant pores of 1.3 nm and some large pores in the mesoporous region (2–10 nm). In more detail, the mesoporous size distribution of PAF-201 was evidently different from that of PAF-200, also suggesting the occurrence of framework deformation. In addition, we also prepared another triphenylcarbinol-based material (TAF–OH) by reductive coupling of tri(4-bromobenzene)carbinol, as reported previously by Comotti *et al.*^16^ However, this framework could not be transformed into the tritylium-based one, possibly due to its rigidity.

The Fourier transform infrared (FT-IR) spectrum of PAF-200 exhibited an O–H band at 3500 cm^−1^ and a C–O band at 1069 cm^−1^ (Fig. S2†), indicating that this framework retained the tertiary alcohol group of the substrate. The observation of a saturated hydrocarbon C–H band at 3000–2700 cm^−1^ and a C–Cl band at 775 cm^−1^ showed that chloroform molecules were involved in the framework formation as cross-linkers and a Friedel–Crafts reaction took place. This was further supported by the chlorine contents of 6.13 wt% and 6.08 wt% in the two PAFs, respectively (Table S2†). The Friedel–Crafts reaction could introduce CHCl_2_ units at the end of the framework. In contrast to those of PAF-200, the O–H band became very weak and the B–F characteristic peak at 1083 cm^−1^ emerged in the IR spectrum of PAF-201, suggesting the formation of tritylium units in PAF-201. Furthermore, in the solid-state ^13^C CP/MAS NMR spectrum of PAF-201, the signal at 81 ppm belonging to the tertiary C–OH significantly weakened as compared to that for PAF-200 (Fig. 1c and d), and a strong signal at 190 ppm belonging to carbonium was observed. The signal peaks of three kinds of cross-linkers, CH, CHCl and CHOH, were also observed at 57 ppm, 69 ppm and 74 ppm, respectively. This indicated that the chloride atoms of CHCl_3_ molecules as a reaction substrate were not completely depleted. However, the resultant imperfect framework may endow itself with moderate flexibility to withdraw the tension caused by molecular deformation. Notably, mild activation of PAF-201 was applied because the tritylium units were easily destroyed at higher temperature, and thus a small amount of residual THF molecules in PAF-201 was observed from IR and NMR spectra (Fig. 1d and S2†). According to the BF_4_^−^ content determined by inductively coupled plasma atomic emission spectrometry element analysis, the amount of carbonium in PAF-201 was determined to be about 0.92–1.14 mmol g^−1^. Powder X-ray diffraction patterns of the two PAFs indicated that they had no long-range ordered structures (Fig. S3†). Thermogravimetric analysis showed that they began to decompose above 350 °C under air atmosphere (Fig. S4†). Transmission electron microscopic and scanning electron microscopic images of the two PAFs displayed that they were solid spheres with diameters of 200 nm to 1.0 μm (Fig. S5†).

### Response of PAF-200 to acid gas

**Fig. 2:**
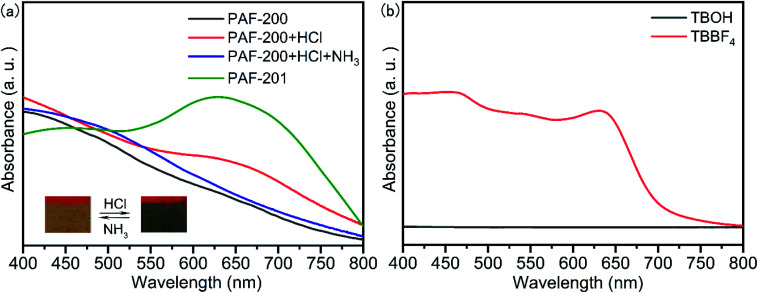
Solid visible absorption spectra of PAF-200 sample exposed to HCl and NH_3_ gas, alternately, and PAF-201 (a), and TBOH and TBBF_4_ (b). The insets show the allochroic photographs of the PAF-200 sample.

The transformation of TBOH to tri(4-biphenyl)carbonium can occur in acidic solution with a distinct color change. The addition of a small amount of conc. HCl to TBOH in CH_3_CN resulted in a strong adsorption at around 540 nm (Fig. S6†). Interestingly, when TBOH solid was exposed to HCl gas, this allochroic phenomenon could not be observed, possibly due to compact molecular packing. We further explored whether PAF-200 could respond to acid gas or not. As shown in the video (see ESI†), the color of PAF-200 was changed from earthy yellow to dark blue in <1 s after being exposed to HCl gas. A new absorption band appeared at around 650 nm in the solid visible absorption spectrum of PAF-200, the same as that of tri(4-biphenyl)carbonium tetrafluoroborate (TBBF_4_) (Fig. 2). The color of this material was further changed to earthy yellow in <1 s after being exposed to NH_3_/H_2_O gas owing to the formation of triphenylmethylamine/triphenylcarbinol units. This allochroic cycle can be repeated at least fifty times. Especially, all the C–OH units of PAF-200 were transformed into C–H units under reflux in HCOOH solution, and the resulting PAF-200H framework did not respond to HCl gas (Fig. S7†). The above results indicate that some of the tritylium units on the framework could react with the donor substrate repeatedly. This meant that the tritylium ion at the node of PAF-201 could potentially be used as a Lewis acid catalyst because the catalytic process is also along with a deformation of tritylium between plane and tetrahedron.

### Lewis acid catalytic performance of PAF-201

**Fig. 3:**
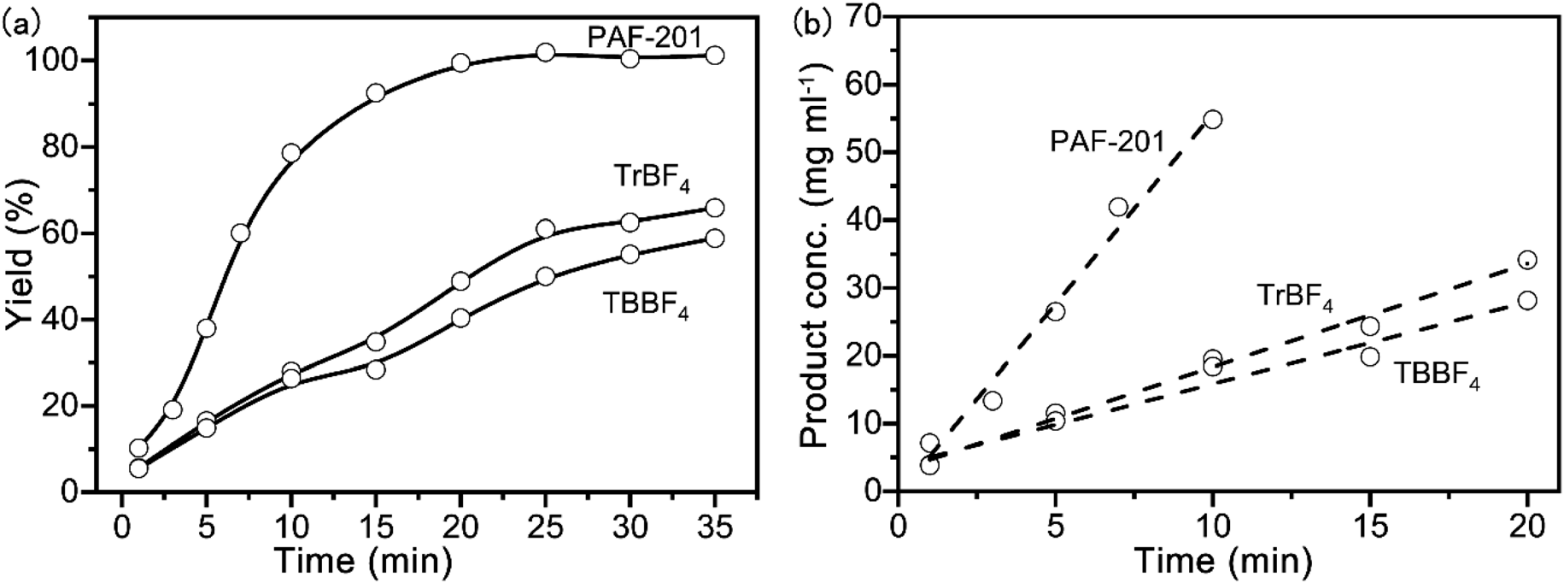
Under pseudo first-order kinetic conditions, the yield *versus* time curves of a Povarov reaction using different catalysts (a) and linear fitting for initial reaction rates (b). Reaction conditions: catalyst (0.005 mmol), 2,3-dihydrofuran (10 mmol), benzylidene aniline (2 mmol), and anhydrous THF (5 ml) at room temperature.

The Povarov reaction is one of the most efficient strategies to synthesize drug intermediate tetrahydroquinoline derivatives.^17^ In order to evaluate the Lewis acid catalytic performance of PAF-201, the Povarov reaction was selected as the model reaction. First, 2,3-dihydrofuran and benzylidene aniline were reacted in anhydrous tetrahydrofuran at room temperature with different PAF-201 loadings (Table 1, entries 1–4). PAF-201 loading of only 0.25 mol% (based on carbonium) gave the product with an isolated yield of 92% in one hour. In contrast, tritylium tetrafluoroborate (TrBF_4_) and TBBF_4_ at 0.25 mol% only afforded isolated yields of 74% and 67% in three hours (Table 1, entries 5–6), respectively. Under pseudo-first-order kinetic conditions (Fig. 3), the initial reaction rates were 5.62 mg ml^−1^ min^−1^ for PAF-201, 1.52 mg ml^−1^ min^−1^ for TrBF_4_ and 1.20 mg ml^−1^ min^−1^ for TBBF_4_. PAF-201 evidently exhibited more efficient catalytic activity than TrBF_4_ or TBBF_4_. However, the use of 0.5 mol% TrBF_4_ gave 92% yield (Table 1, entry 7). Furthermore, using the Schiff bases with Br or OCH_3_ substituents as substrates, PAF-201 also gave excellent isolated yields of 85–96%, better than the yield with TrBF_4_ (Table 1, entries 8–15). In order to testify that it is triphenylcarbonium, not Brønsted acid, that catalyzes this Povarov reaction, a control experiment was carried out. 2,6-Di-*tert*-butylpyridine (DBPy) at 1 mol% as a proton scavenger was added into the Povarov reaction system (Table 1, entry 16).^18^ As expected, the resultant yield of 89% was almost comparable to that without the proton scavenger. Notably, almost no product was obtained when the concentrations of the reaction substrates were decreased to one tenth of the original concentrations (Table 1, entry 17). This suggested that the enrichment of porous material to the reaction substrate had little influence on this reaction.

**Table 1:** The Povarov reaction using different triarylcarbenium catalysts^a^

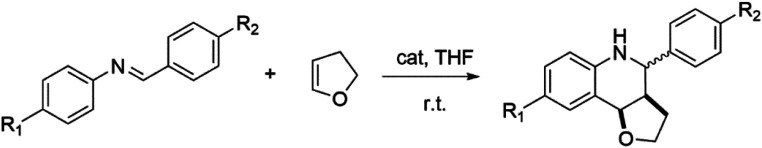					
Entry	Schiff base	Catalyst	Time [h]	Isolated yield [%]	Dr [*cis* : *trans*]^b^
1	R_1_ = H, R_2_ = H	PAF-201^c^	1	72	49 : 51
2	R_1_ = H, R_2_ = H	PAF-201	1	91	49 : 51
3	R_1_ = H, R_2_ = H	PAF-201^d^	0.75	90	49 : 51
4	R_1_ = H, R_2_ = H	PAF-201^e^	0.5	92	49 : 51
5	R_1_ = H, R_2_ = H	TrBF_4_^f^	3	74	49 : 51
6	R_1_ = H, R_2_ = H	TBBF_4_^f^	3	67	50 : 50
7	R_1_ = H, R_2_ = H	TrBF_4_	1	90	49 : 51
8	R_1_ = Br, R_2_ = H	PAF-201	1	90	52 : 48
9	R_1_ = Br, R_2_ = H	TrBF_4_	1	83	43 : 57
10	R_1_ = H, R_2_ = Br	PAF-201	1	92	47 : 53
11	R_1_ = H, R_2_ = Br	TrBF_4_	1	85	34 : 66
12	R_1_ = OMe, R_2_ = H	PAF-201	1	85	61 : 39
13	R_1_ = OMe, R_2_ = H	TrBF_4_	1	84	55 : 45
14	R_1_ = H, R_2_ = OMe	PAF-201	1	86	61 : 39
15	R_1_ = H, R_2_ = OMe	TrBF_4_	1	79	57 : 43
16^g^	R_1_ = H, R_2_ = H	PAF-201	2	89	48 : 52
17^h^	R_1_ = H, R_2_ = H	PAF-201	2	—i	—
18	R_1_ = H, R_2_ = H	PAF-201^j^	1	92	49 : 51
19	R_1_ = H, R_2_ = H	PAF-201^k^	1	91	49 : 51
20	R_1_ = H, R_2_ = H	PAF-201^l^	1	92	49 : 51

a Reaction conditions: 2,3-dihydrofuran (4.0 mmol), Schiff base (2.0 mmol), anhydrous THF (5 ml), PAF-201 (0.25 mol%), TBBF_4_ (0.5 mol%) or TrBF_4_ (0.5 mol%) used unless otherwise specified.

b Diastereomeric ratio, determined by NMR analyses.

c 0.1 mol% used.

d 0.45 mol% used.

e 0.68 mol% used.

f 0.25 mol% used.

g 1 mol% 2,6-di-*tert*-butylpyridine used.

h 50 ml of anhydrous THF.

i Trace product monitored by HPLC.

j First cycled PAF-201 by oxidation and acidification.

k Second cycled PAF-201 by oxidation and acidification.

l Third cycled PAF-201 by oxidation and acidification.

After completion of the Povarov reaction, the PAF-201 catalyst was easily recycled by filtration separation, then KO*t*-Bu treatment in DMSO under air atmosphere and subsequent HBF_4_ acidification. The oxidation process can effectively transform a small amount of tritan units into tritanol ones. The carbonium content of the regenerated PAF-201 was still ∼0.98 mmol g^−1^. This recycled PAF-201 could be applied to the model reaction again. The process was repeated at least three times and the isolated yields of this reaction were almost unchanged (Table 1, entries 18–20). The FTIR and solid-state ^13^C CP/MAS NMR spectra of fresh and regenerated PAF-201 showed no obvious differences (Fig. S9 and S10†).

### Hydride-abstracting performance of PAF-201

Tritylium ion can be used as a hydride abstractor in organic synthesis.^19^ We further tested the hydride-abstracting performance of PAF-201 using tropilidene as the substrate. The equivalent of PAF-201 (based on carbonium) can completely transform tropilidene into tropolium tetrafluoroborate in anhydrous CH_3_CN at 0 °C. However, this oxidation using PAF-201 was completed in twelve hours, and only two hours for TrBF_4_. It was time-assuming that all the active tritylium units on the framework were transformed into tritan ones with the big framework deformation. The solid-state ^13^C CP/MAS NMR spectrum of the recycled PAF-201 sample showed the strengthened signal at 55 ppm, indicating that all carboniums were converted into C–Hs (Fig. S11†).

### Catalytic mechanism of PAF-201 in Povarov reaction

**Fig. 4:**
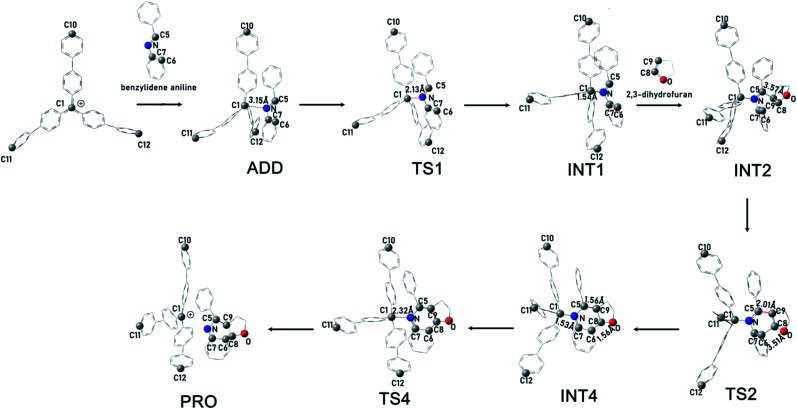
Schematic diagram of intermediates and transition species of a Povarov reaction using C10, C11, C12-fixed tri(4-biphenyl)carbonium (representing PAF-201) as the catalyst and benzylidene aniline and 2,3-dihydrofuran as the substrate. Hydrogen atoms are omitted for clarity. C, gray; N, blue; O, red.

**Fig. 5:**
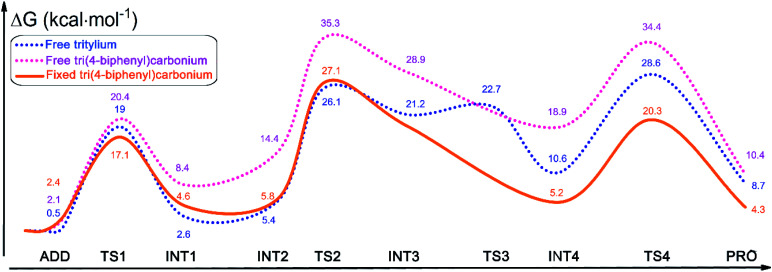
Calculated free energy profiles for the Povarov reaction with *N*-benzylideneaniline and 1,2-dihydrofuran as the substrate, and tritylium (blue), free (pink) or fixed (orange) tri(4-biphenyl)carbonium as the catalyst.

To rationalize the rate acceleration of the Povarov reaction observed with PAF-201 relative to TrBF_4_ or TBBF_4_, DFT calculations were undertaken. The free energy profiles of the Povarov reaction catalyzed by PAF-201, TrBF_4_ or TBBF_4_, and corresponding reaction pathways are shown in Fig. 4, 5 and S12, S13.† When triphenylcarbinol units in PAF-200 were dehydroxylated into tritylium ones in PAF-201, the tetrahedral conformation of the carbon-centered structure will tend to be transformed into a planar one, as in free TrBF_4_ or TBBF_4_. However, this transformation could be constrained by the remaining framework. To consider the constraint of the surrounding framework, we fixed three end carbon atoms of tri(4-biphenyl)carbonium and tritylium units in the optimization. Thus, two cluster models, fixed tri(4-biphenyl)carbonium and tritylium, were used to represent the catalytic site of PAF-201. Their construction details are described in the ESI. As observed from Fig. 4, the Povarov reaction catalyzed by tritylium ion can be divided into four steps: (1) the activation of benzylidene aniline *via* Lewis acidic tritylium ion to form intermediate INT1; (2) the [4 + 2] cycloaddition reaction between the activated INT1 and the electron-rich alkene 2,3-dihydrofuran with the concerted or step-wise pathways; (3) the dissociation of the cycloaddition product INT4 and the regeneration of the carbonium; and (4) the tautomerization (1,3-H shift) of the released product PRO. The free energy barrier of the fixed case (representing PAF-201) in each step of the Povarov reaction is lower than those for the free cases. We also noted that the free energy barriers of the former three reaction steps in the fixed tri(4-biphenyl)carbonium are lower than those in the free tritylium by 1.9 kcal mol^−1^, 1.0 kcal mol^−1^ and 2.9 kcal mol^−1^, respectively. These calculated results suggest that PAF-201 is a better Lewis acid catalyst than free tritylium and tri(4-biphenyl)carbonium, in agreement with the pseudo-first-order reaction rates.

**Fig. 6:**
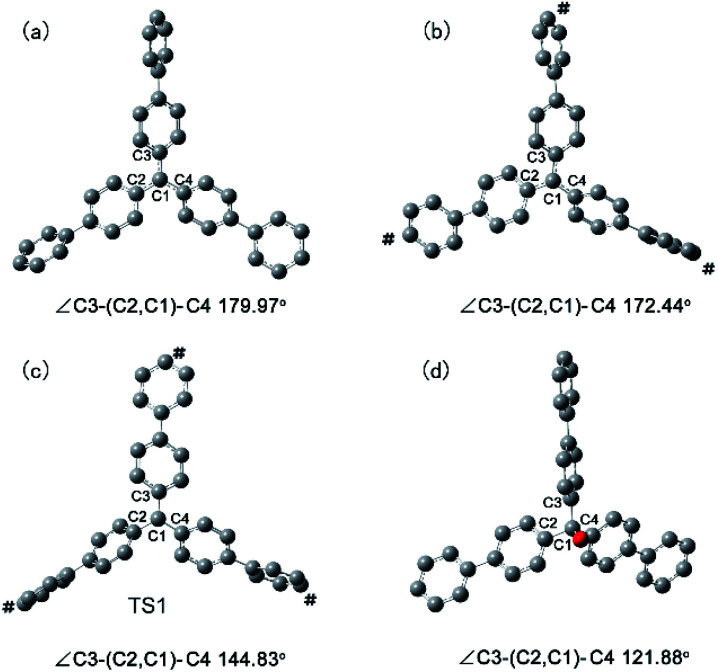
The optimized minimum energy structures of free (a) and fixed (b) tri(4-biphenyl)carbonium, and TS1 of fixed tri(4-biphenyl)carbonium interacting with imine (c) and tri(4-biphenyl)carbinol (d). ∠C3–(C2, C1)–C4 denotes the dihedral angle. The sign # marks the fixed end carbon atoms.

To gain deeper insight into the origin of the high Lewis acid catalytic activity of PAF-201, we focused more closely on the geometric details of tri(4-biphenyl)carbinol and free and fixed tri(4-biphenyl)carbonium (Fig. 6). In free tri(4-biphenyl)carb-onium, the carbonium atom (C1) and three carbon atoms (C2, C3, C4) connecting with it are co-planar with the dihedral angle ∠C3–(C2, C1)–C4 of 179.97° (planar). From tri(4-biphenyl)carb-inol to fixed tri(4-biphenyl)carbonium, the ∠C3–(C2, C1)–C4 value was changed from 121.88° to 172.44° (quasi-planar). Notably, the energy of the fixed tri(4-biphenyl)carbonium was 3.7 kcal mol^−1^ higher than that of the free one, indicating the existence of tension in the fixed structure. Thus, the PAF-201 framework was taut so as to reserve the rebound force for reforming the tetrahedral structure. This is favourable for tritylium to activate benzylidene aniline in the Povarov reaction along with a deformation of the quasi-plane to a tetrahedron. In addition, the quasi-planar structure is closer to the TS1 structure with the ∠C3–(C2, C1)–C4 angle of 144.83° than the perfect one. This means that a smaller distortion energy is required from the reactant to the TS1 as activating benzylidene aniline. As a consequence, we could conclude that the quasi-planar tritylium ion at the node of the tensile PAF-201 framework had better Lewis acid catalytic activity.

## Experimental

### Synthesis of PAF-200 (ref. 20)

A 250 ml round-bottomed flask with anhydrous AlCl_3_ (2.5 g) was evacuated and inflated with Ar three times. Then CHCl_3_ (120 ml) was injected into the flask *via* a syringe, and this resultant system was stirred for 1 h at 60 °C. Furthermore, a solution of tri(4-biphenyl)carbinol (1.0 g) in CHCl_3_ (30 ml) was added into the flask. The colour of the reaction system immediately changed from light yellow to purple. The mixture was stirred for 24 h at 60 °C. The resulting dark blue powder was collected by filtration, subsequently washed with 1 M HCl solution and water repeatedly, further purified by Soxhlet extraction successively with methanol, THF and CH_2_Cl_2_ for 36 h, and then dried at 120 °C for 12 h under vacuum to give the earthy yellow powder product (1.2 g).

### Synthesis of PAF-201 (ref. 21)

A 100 ml round-bottomed flask with PAF-200 (2.0 g) was evacuated and inflated with Ar three times. HBF_4_–Et_2_O (52 wt%, 20 ml) was injected into the flask *via* a syringe at 0 °C and this reaction system was kept at 25 °C for 24 h. The raw powder was collected by centrifugation, repeatedly washed with anhydrous THF, and dried for 12 h under vacuum at 25 °C, to give the dark blue powder product (2.0 g).

The characterization details of the PAF materials are provided in the ESI.†

## Conclusions

To summarize, we have successfully prepared two new porous aromatic frameworks, triphenylcarbinol-based PAF-200 and tritylium-based PAF-201. Some of the triphenylcarbinol units of PAF-200 were transformed into tritylium ions, affording the taut PAF-201 framework. A fast allochroic cycle was observed when PAF-200 was alternately exposed to HCl and NH_3_ gas. PAF-201 exhibited better Lewis acid catalytic activity than TrBF4 and TBBF_4_. The theoretical computational results showed that the quasi-planar tritylium ion as a node of a tensile network could more easily interact with the imine substrate and also dissociate with the product. PAF-201 as a Lewis acid catalyst could be well recycled for at least three times without evident loss of catalytic activity. Moreover, PAF-201 was also used as a dehydrogenative reagent in transforming tropilidene into tropolium. The further application of this tensile framework is underway in our laboratory.

## Data availability

Some data has been provided in ESI.

## Author contributions

Z. B. and G. Z. initiated and designed this work. Z. B. and F. C. wrote this paper. S. Z. conducted the synthesis and characterization of PAFs. J. Z. and F. C. conducted the computational work. Y. Z. conducted partial characterization work. X. Z. and S. W. revised this paper.

## Conflicts of interest

There are no conflicts to declare.

## Supplementary Material

SC-012-D1SC02594E-s001

SC-012-D1SC02594E-s002
