# Supplementary material for: Highly efficient Lewis acid catalytic activity of the tritylium ion at the node of a tensile organic framework
Source: Chem Sci. 2021 Jun 11;12(28):9786–93. doi: 10.1039/d1sc02594e (PMC8293798; doi:10.1039/d1sc02594e)
Supplement: SC-012-D1SC02594E-s001 [file SC-012-D1SC02594E-s001.pdf]

Electronic Supplementary Material (ESI) for Chemical Science.
This journal is © The Royal Society of Chemistry 2021

Supplementary Information

Highly efficient Lewis acid catalytic activity of tritylium ion at the node of tensile organic framework

Shuai Zhao, Juhui Zhang, Yongchang Zhai, Xiaoqin Zou, Shaolei Wang, Zheng Bian,* Fengchao Cui,* Guangshan Zhu*

Contents

1 General
2 Synthesis of tri(4-biphenyl)carbonium tetrafluoroborate
3 The Friedel-Crafts product with triphenylcarbinol as substrate
4 Characterization of PAF-200 and PAF-201
5 The spectra response of TBOH in CH_3CN to acid
6 Tri(4-biphenyl)methane-based porous material PAF-200H
7 Povarov reaction with PAF-201, $TBBF_4$ or $TrBF_4$ as catalysts
8 The recycle of PAF-201
9 Hydride-abstracting reaction of PAF-201
10 Computational details
11 ^1H NMR spectra of organic compounds

1. General

All moisture or oxygen-sensitive reactions were carried out under an argon atmosphere in oven or heat-dried flasks. The solvents were purified by distillation over the drying agents indicated and were transferred under argon: Et_2O (Na), CH_2Cl_2 (CaH_2), THF (Na), $CHCl_3$ (4Å sieve). All reactions were monitored by thin-layer plates using UV light as visualizing agent (if applicable). The products were purified by flash column chromatography on silica gel (200-300 meshes) from the Qingdao Marine Chemical Factory in China. ^{1}H NMR spectra were recorded in $CDCl_3$ or CD_3CN solution on a Varian 400 MHz instrument or a Bruker 500 MHz instrument. Chemical shifts were denoted in ppm, and calibrated by using solvent $CDCl_3$ (7.26 ppm), CD_3CN (1.96 ppm)) as internal reference for ^{1}H NMR. Solid-state ^{13}C CP/MAS NMR spectra were obtained using a Bruker Avance III model 400 MHz NMR spectrometer at a MAS rate of 5 kHz. FT-IR measurements were performed on the Nicolet IS50 Fourier transforms infrared spectrometer. TGA were measured on the METTLER-TOLEDO TGA/DSC 3+ analyzer at the 10 °C min^{-1} heating rate in air atmosphere. The Elemental analyses (for C and H) were measured using a Perkin Elmer 2400 Series II CHNS/O Analyzer. The Elemental analyses (for Al, B, F and Cl) were measured by Inductively Coupled Plasma - Atomic Emission Spectrometer (ICP-AES) on a LEEMAN Prodigy. N_2-adsorption isotherms and pore size distribution were obtained at 77 K using an Autosorb iQ2 adsorptometer, Quantachrome Instrument. Powder X-ray diffraction (PXRD) was performed by a Riguku D/MAX2550 diffractometer using Cu-Kα radiation, 40 kV, 200 mA with a scanning rate of 8 °min^{-1} (2ϑ). Transmission electron microscopic (TEM) images were recorded using a JEOL JEM 3010 instrument with an acceleration voltage of 300 kV. Scanning electron Microscopy (SEM) analysis was performed on a JEOS JSM 6700 system.
All chemicals are used directly from commerce without otherwise specified.

2. Synthesis of tri(4-biphenyl)carbonium tetrafluoroborate

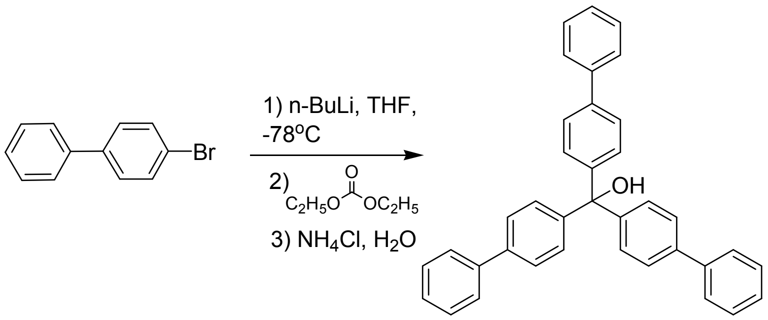

Tri(4-biphenyl)carbinol: [1] 4-Bromodiphenyl (2.80 g, 12 mmol) and anhydrous THF (50 ml) were added to a 2-neck round-bottom flask under Ar atmosphere. After cooled down to -78 °C, a 2.5 M hexane solution of *n*-BuLi (4.6 ml, 12 mmol) was slowly added into this solution. After stirring for 3 h, diethyl carbonate (0.47 g, 4 mmol) was added dropwise to this system. Then, the solution was slowly warmed up to room temperature. After stirring for 6 h, the reaction system was quenched with NH_4Cl

saturated aqueous solution (50 ml) and extracted with CH_2Cl_2 (3 x 50 ml). The organic phase was collected, washed with brine and water, and dried with anhydrous Na_2SO_4. After removal of the solvent under reduced pressure, the residue was purified by silica gel column chromatography (petroleum ether/dichloromethane, 5/2, v/v) to give target compound. Yield (1.54 g, 79%). ^1H NMR (500 MHz, ppm, $CDCl_3$): δ =7.67-7.58 (m, 12 H), 7.46-7.43 (m, 12 H), 7.37-7.34 (m, 3 H), 2.90 (s, 1 H).

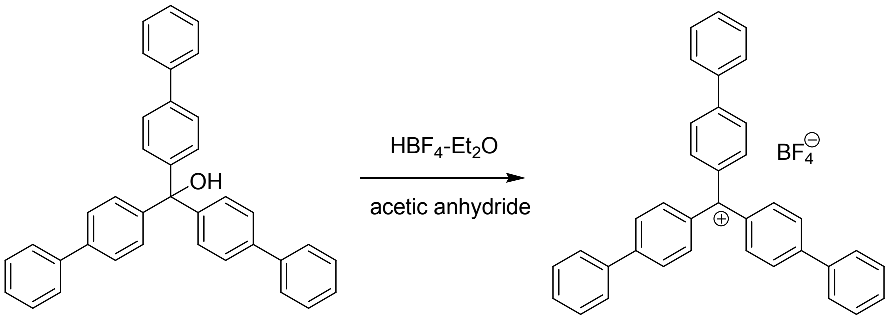

Tri(4-biphenyl)carbonium tetrafluoroborate:[2] To a suspension of tri(4-biphenyl)carbinol (100 mg, 0.205 mmol) in acetic anhydride (2 ml) was added several drops of HBF_4-Et_2O (50-55 wt%). The resultant solution was kept at 4 ºC until deep red crystal was formed. ^1H NMR (500 MHz, ppm, $CDCl_3$): δ = 8.13-8.10 (d, 6H), 7.89-7.80 (d, 12H), 7.63-7.59 (m, 9H); ^{13}C NMR (125 MHz, ppm, $CDCl_3$) 200.28, 154.96, 141.89, 138.40, 137.28, 131.48, 129.79, 128.78, 128.15.

3. The Friedel-Crafts product with triphenylcarbinol as substrate

A 250 ml round-shaped flask with anhydrous $AlCl_3$ (0.20 g) was evacuated and inflated with Ar for 3 times. Then $CHCl_3$ (25 ml) was injected into the flask via a syringe. This resultant system was kept for 1 h at 60 ^oC. Furthermore, triphenylcarbinol (0.10 g) in $CHCl_3$ (8 ml) was added into the flask. The mixture was stirring for 24 h at 60 ^oC. The black powder was collected by filtration, subsequently washed with 1 M HCl and water, respectively, further purified by Soxhlet extraction successively with THF and CH_2Cl_2 for 36 h, and subsequently dried at 120 ^oC for 12 h in vacuum, to give the dark brown powder product (47 mg).

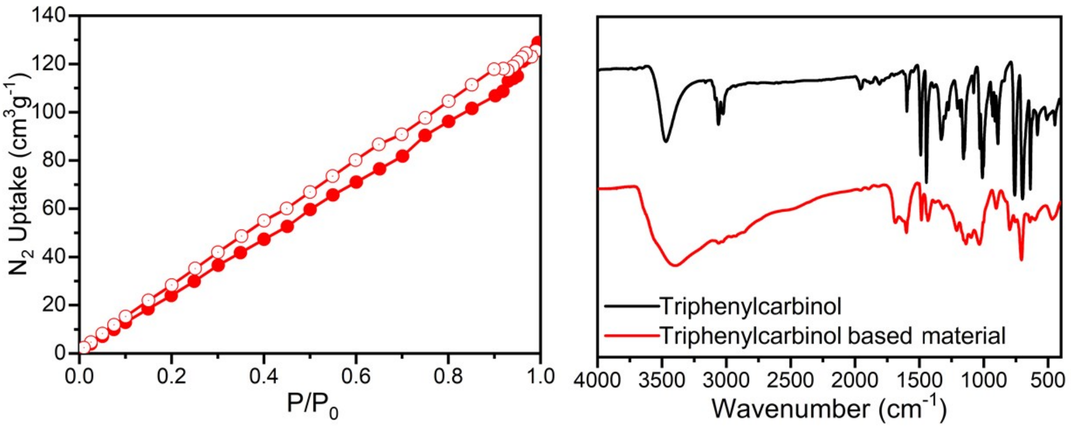

Fig. S1 N_2 adsorption and desorption isotherms at 77 K (left) and FT-IR spectra of

triphenylcarbinol-based material (right).

4. Characterization of PAF-200 and PAF-201

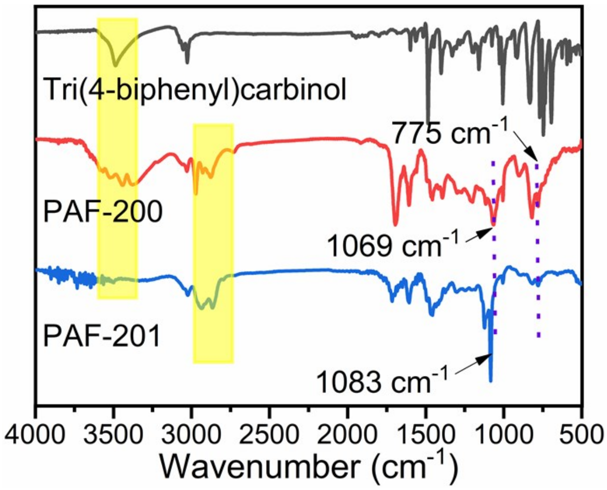

Fig. S2 FT-IR spectra of tri(4-biphenyl)carbinol (black), PAF-200 (red) and PAF-201 (blue).

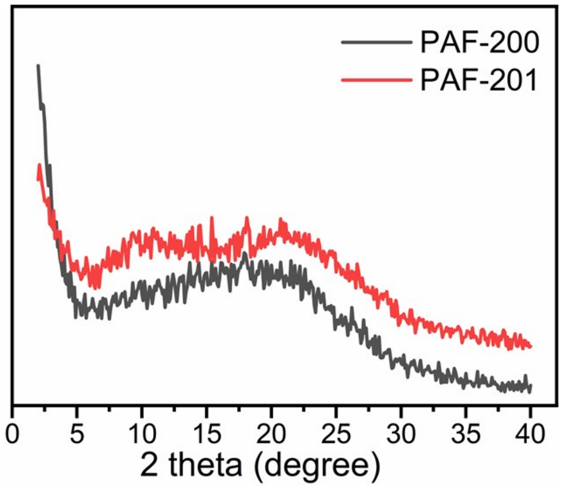

Fig. S3 PXRD patterns of PAF-200 and PAF-201 materials.

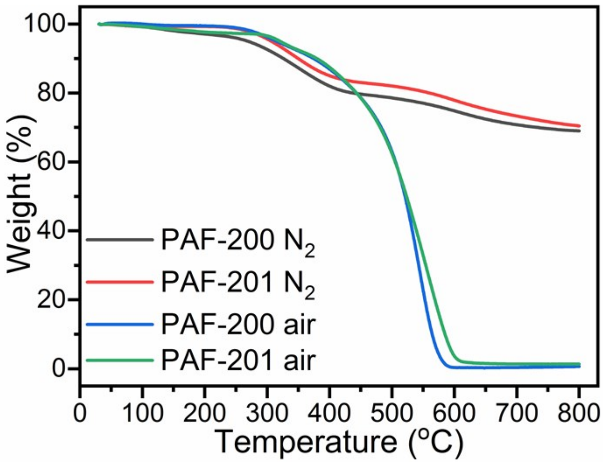

Fig. S4 TGA curves of PAF-200 and PAF-201 under N_2 and air atmosphere.

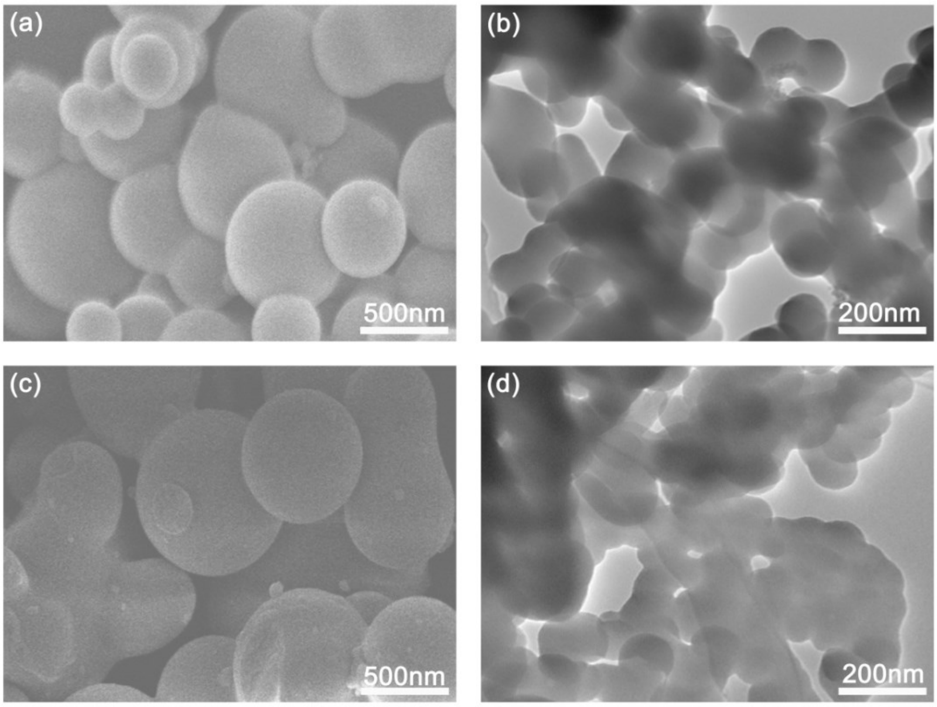

Fig. S5 SEM (left) and TEM (right) images of PAF-200 (a-b) and PAF-201 (c-d). Scale bars: a, c, 500 nm; b, d, 200 nm.

Table S1. Textural data for PAF-200 and PAF-201

sample	S_{BET}[a)] [$m^2\,g^{-1}$]	Micropore area [$m^2\,g^{-1}$]	External surface area [$m^2\,g^{-1}$]	V_{total}[b)] [$cm^3\,g^{-1}$]	V_{micro}[c)] [$cm^3\,g^{-1}$]	V_{meso} [$cm^3\,g^{-1}$]
PAF-200	1234.87	684.71	552.16	0.78	0.30	0.48
PAF-201	917.45	501.71	401.74	0.59	0.23	0.36

[a)] BET surface areas, calculated at P/P_0 = 0.005-0.05;
[b)] Total pore volumes, calculated at P/P_0 = 0.98;
[c)] Cumulative pore volumes, calculated at pore width ≤ 2.0 nm (micropore volumes).

Table S2. Elemental analysis results of PAF-200 and PAF-201

sample	%C	%H	%Al	%Cl	%B [a]	%F
PAF-200	67.391	8.103	0.3791	6.13	\	\
PAF-201	60.484	7.57	0.3519	6.08	1.2370	8.41

[a] By ICP method. Before analysis, ~ 15 mg of sample was digested in nitromuriatic acid (10 mL) and transferred to a 50 mL volumetric flask with distilled water.

5. The spectra response of TBOH in CH_3CN to acid

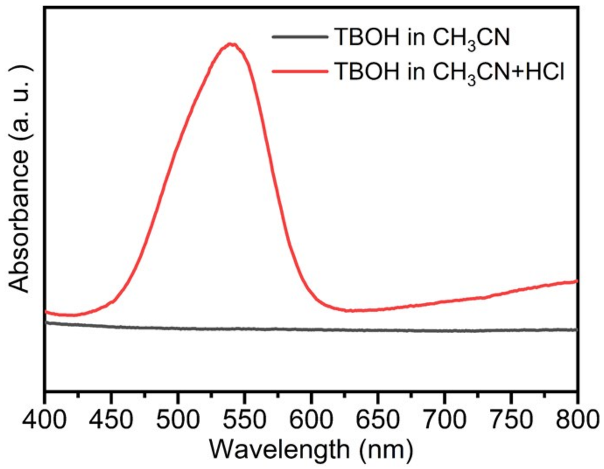

Fig. S6 The visible absorption spectra of TBOH in CH_3CN before and after dropping a drop of concentrated HCl at room temperature. *c*, 1.0 mg/mL.

6. Tri(4-biphenyl)methane-based porous material PAF-200H [3]

Under Ar atmosphere, the mixture of PAF-200 (200 mg) and formic acid (20 ml) was heated at 90 °C for 12 hours. PAF-200H was obtained by filtration, washed with water (100 ml × 3), methanol (100 ml × 3) and under vacuum dried at 100 °C for 12 h.

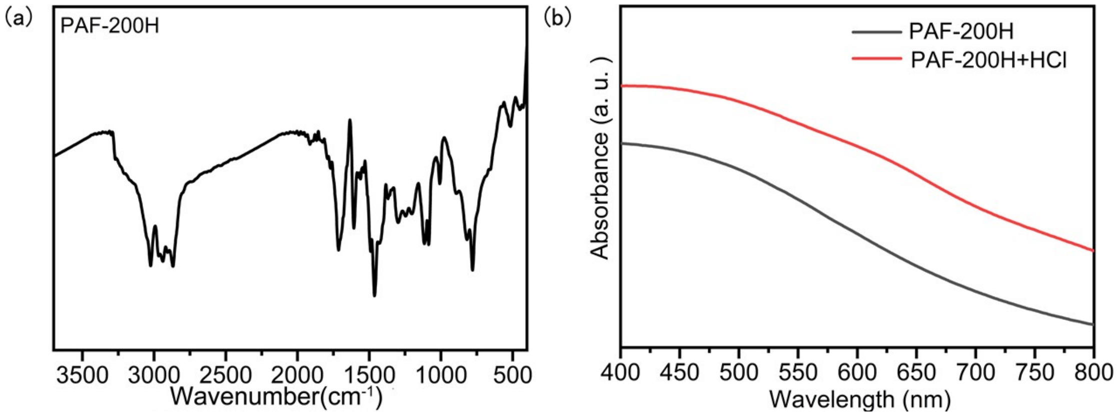

Fig. S7 (a) IR spectra of PAF-200H; (b) UV-vis absorption spectra of PAF-200H before and after exposed to HCl gas.

7. Povarov reaction with PAF-201, $TBBF_4$ or $TrBF_4$ as catalysts

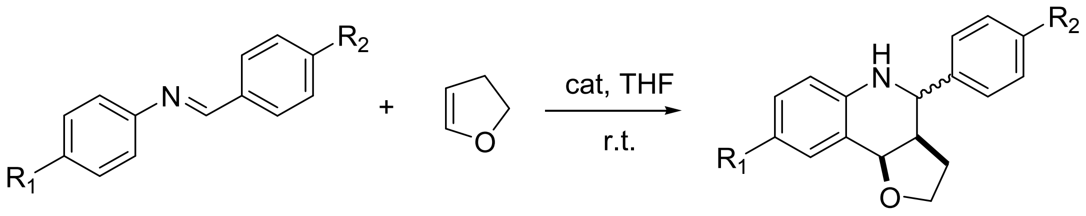

Typically, Schiff base (2 mmol) and catalyst were added to a dried round-shaped flask. This resultant system was evacuated and inflated with Ar for 3 times. Then anhydrous THF (5 ml) and 2,3-dihydrofuran (4 mmol) were added by a syringe. The mixture was stirred at room temperature. The reaction process was monitored by TLC. The crude product was separated by centrifugation and further purified through column chromatography on silica gel.

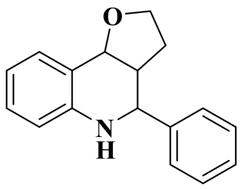

4-Phenyl-2,3,3a,4,5,9b-hexahydrofuro[3,2-c]quinoline (**1**): ^{1}H NMR (400 MHz, ppm, $CDCl_3$): δ 7.48-7.44 (m, 2H), 7.41-7.29 (m, 4H), 7.15-7.08 (m, 1H), 6.83-6.79 (t, *J* = 7.4 Hz, 1H), 6.61 (d, *J* = 8.0 Hz, 1H), 5.29 (d, *J* = 8.0 Hz, 0.5H), 4.70 (d, *J* = 2.9 Hz, 1H), 4.61 (d, *J* = 5.2 Hz, 1H), 4.19 (br, 1H), 4.07-4.01 (m, 1H), 3.85-3.80 (m, 2H), 3.76-3.69 (m, 1H), 2.83-2.76 (m, 1H), 2.50-2.43 (m, 1H), 2.27-2.17 (m, 1H), 2.05-1.98 (m, 1H), 1.76-1.69 (m, 1H), 1.57-1.49 (m, 1H).

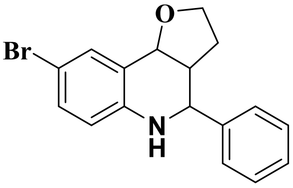

8-Bromo-4-phenyl-2,3,3a,4,5,9b-hexahydrofuro[3,2-c]quinolone (**2**): ^{1}H NMR (400 MHz, ppm, $CDCl_3$): δ 7.45-7.35 (m, 6H), 7.04-7.02 (m, 1H), 6.53 (d, *J* = 8.4 Hz, 1H), 5.21 (d, *J* = 8.4 Hz, 0.5H), 4.69 (d, *J* = 2.8 Hz, 1H), 4.05-3.99 (m, 1H), 3.87-3.81 (m, 2H), 3.76-3.69 (m, 1H), 2.80-2.76 (m, 1H), 2.47-2.41 (m, 1H), 2.22-2.11 (m, 1H), 2.07-1.98 (m, 1H),1.76-1.68 (m, 1H), 1.57-1.50 (m, 1H).

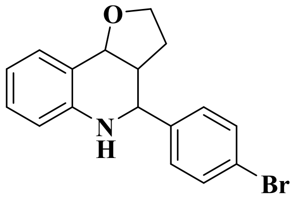

4-(4-Bromo)-2,3,3a,4,5,9b-hexahydrofuro[3,2-c]quinolone (**3**): ^{1}HNMR (400 MHz, ppm, $CDCl_3$): δ 7.51-7.33 (m, 5H), 7.13-7.09 (m, 1H), 6.87-6.83 (m, 1H), 6.62 (d, *J* = 7.6 Hz, 1H), 5.27 (d, *J* = 8 Hz, 0.5H), 4.68 (d, *J* = 2.8 Hz, 1H), 3.84-3.79 (m, 1H), 3.76-3.69 (m, 1H), 2.79-2.72 (m, 1H),2.23-2.15 (m, 1H), 1.66-1.54 (m, 2H).

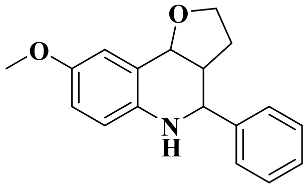

8-Methoxy-4-phenyl-2,3,3a,4,5,9b-hexahydrofuro[3,2-c]quinolone (**4**): ^{1}H NMR (400 MHz, ppm, $CDCl_3$): δ 7.47-7.32 (m, 5H), 6.93 (d, *J* = 2.8 Hz, 1H), 6.73 (dd, *J* = 8.8, 2.8

Hz, 1H), 6.56 (d, J = 8.4 Hz, 1H), 5.25 (d, J = 8 Hz, 0.5H), 4.63 (d, J = 2.8 Hz, 1H), 4.07-3.99 (m, 1H), 3.95 (br, 1H), 3.86-3.80 (m, 1H), 3.78 (s, 3H), 3.74 (d, J = 10.8 Hz, 1H), 3.75-3.69 (m, 1H), 3.64 (s, 1H), 2.82-2.75 (m, 1H), 2.53-2.46 (m, 1H), 2.27-2.17 (m, 1H), 2.06-1.97 (m, 1H),1.75-1.68 (m, 1H), 1.56-1.48 (m, 1H).

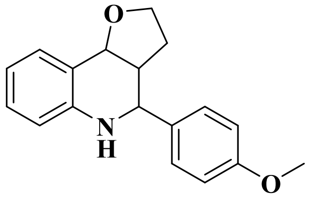

4-(4-Methoxyphenyl)-2,3,3a,4,5,9b-hexahydrofuro[3,2-c]quinolone (**5**): ^{1}H NMR: (400 MHz, ppm, $CDCl_3$) δ 7.43-7.35 (m, 3H), 7.13-7.09 (t, J = 7.6 Hz, 1H), 7.07-6.90 (d, J = 8.8 Hz, 2H), 6.84-6.78 (m, 1H), 6.62-6.57 (m, 1H), 5.26 (d, J = 8.8 Hz, 0.5H), 4.65-4.58 (d, J = 2.8 Hz, 1H), 4.13 (br, 1H), 4.05-3.99 (m, 1H), 3.86-3.80 (m, 4H), 3.75 (d, J = 11.2 Hz, 1H), 2.82-2.73 (m, 1H), 2.46-2.41 (m, 1H),2.27-2.17 (m, 1H), 2.06-1.97 (m, 1H),1.73-1.66 (m, 1H), 1.59-1.56 (m, 1H).

Pseudo first-order reaction kinetics: Benzylidene aniline (2 mmol) and PAF-201 or $TrBF_4$ or $TBBF_4$ (0.25 mol%, based on carbonium) were added to a dried round-shaped flask. This resultant system was evacuated and inflated with Ar for 3 times. Then anhydrous THF (5 ml) and 2,3-dihydrofuran (10 mmol) were added by a syringe. The mixture was stirred at room temperature. At different time intervals, 25 μl aliquot was removed from the reaction system, and diluted in 5 mL of acetonitrile/water (8/2, v/v). The concentrations of resulting samples were analyzed by HPLC. The initial reaction rates were calculated by the linear fitting of concentration-versus-time relationship.

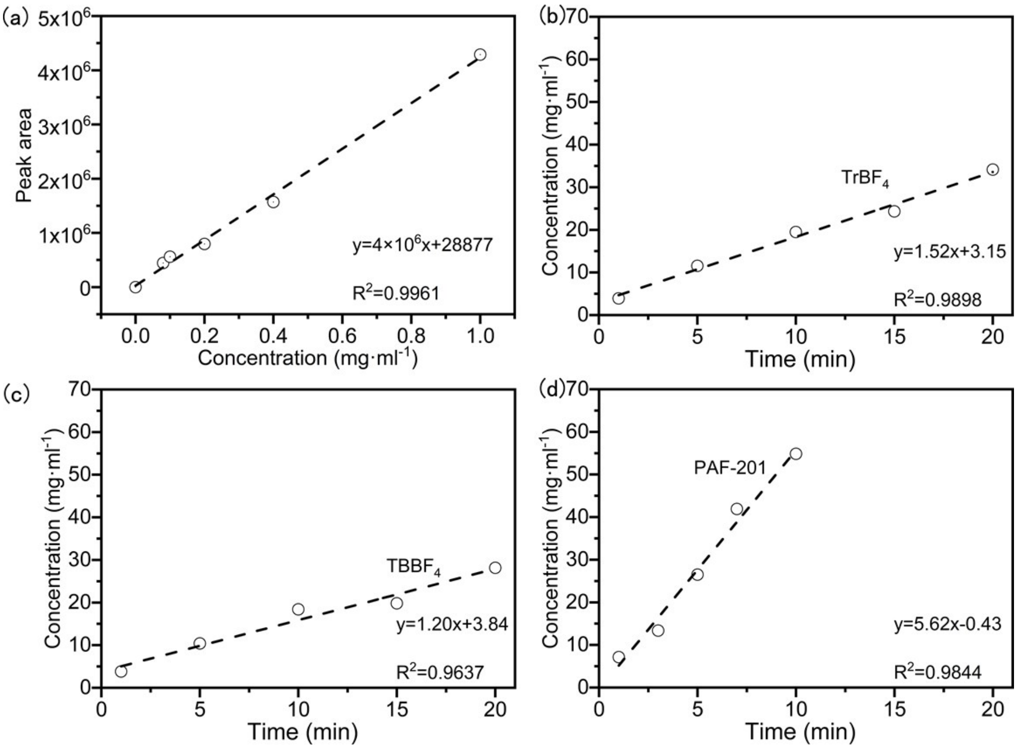

Fig. S8 The standard curve of concentration-versus-HPLC-peak-area of the product

(a), and the linear fitting of pseudo first-order kinetic Povarov reaction using $TrBF_4$ (b), $TBBF_4$ (c) and PAF-201 (d) as catalyst, respectively.

8. The recycle of PAF-201

The PAF powder was separated from reaction system, then stirred with KO*t*-Bu in DMSO under air atmosphere overnight at room temperature, further washed with CH_2Cl_2, dried at 100 ^oC for 5 h, and then added to HBF_4-Et_2O solution (52 wt%). After stirred for 12 h, the suspension was collected by centrifuge, washed by anhydrous tetrahydrofuran, and dried for 12 h at 25 ^oC, to afford the regenerated PAF-201.

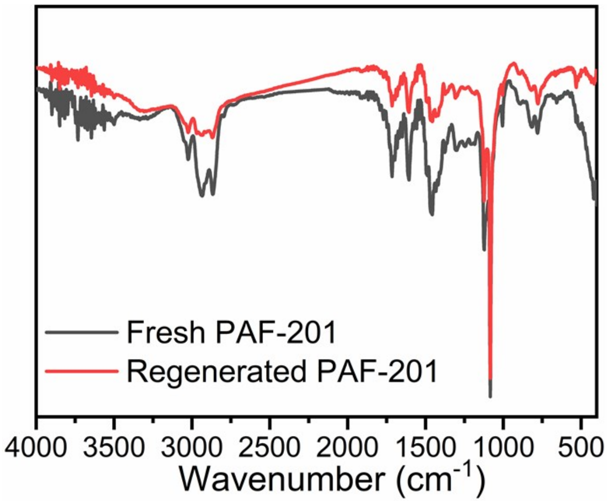

Fig. S9 FT-IR spectra of the fresh and regenerated PAF-201 (used 3 times).

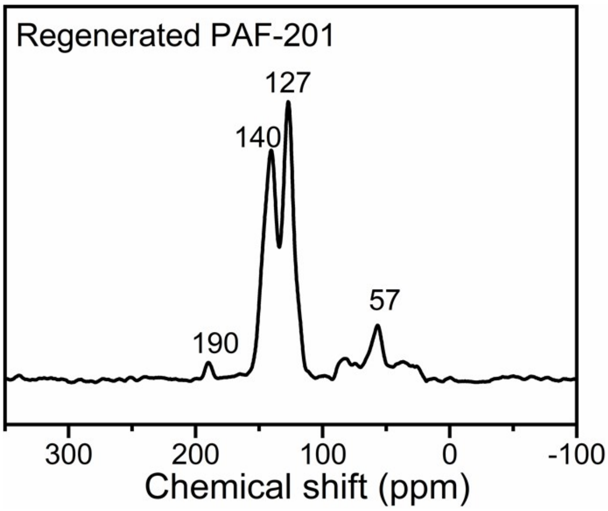

Fig. S10 The ^{13}C CP/MAS NMR spectra of regenerated PAF-201 (used 3 times).

9. Hydride-abstracting reaction of PAF-201

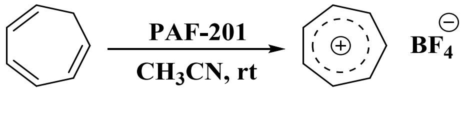

PAF-201 (250 mg, 0.22 mmol), tropilidene (20 mg, 0.22 mmol), and 2 ml dry

acetonitrile were added into a 5 ml bottle. The mixture was stirred for 12 h at room temperature. Then, the PAF solid was separated by filtration. The resultant filtrate was evaporated under reduced pressure and washed with cold dried ether, to give the product of 36 mg (yield: 92%).

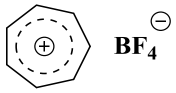

Tropolium tetrafluoroborate: ^{1}H NMR (500 MHz, ppm, CD_3CN): δ = 9.24 (m, 7 H).

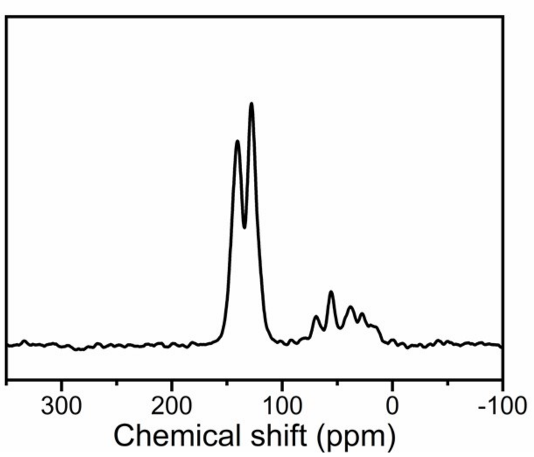

Fig. S11 ^{13}C CP/MAS NMR spectra of the separated PAF material after hydride-abstracting reaction.

10. Computational details

To investigate the Povarov reaction catalyzed by triphenylcarbonium, tri(4-biphenyl)carbonium or PAF-201, we constructed the structural models of triphenylcarbinol, tri(4-biphenyl)methanol, triphenylcarbonium and tri(4-biphenyl)carbonium using GaussView 6.1 version. The geometrical structures of catalytic site of PAF-201 were imitated by removing the hydroxyl group of carbinol and fixing three end carbon atoms of tri(4-biphenyl)carbinol or triphenylcarbinol units in PAF-200. All geometry optimizations were carried out at the M062x level of theory with 6-31G(d, p) basis set for all atoms. Solvent effects are considered with self-consistent reaction cavity continuum solvation model with tetrahydrofuran as solvent [scrf=(cpcm, solvent= tetrahydrofuran)] during the optimization process. Vibrational frequencies were calculated at the same level of theory to confirm the nature of the stationary points (that is, the minima with all real frequencies and the transition states with only one imaginary frequency), and to obtain the thermal corrections to Gibbs free energy at 298 K. To obtain more accurate energies, single-point calculations were done with M062x/6-31++G(2d, 2p) level of theory based on M062x/6-31G(d, p) optimized geometries. For each transition state, intrinsic reaction coordinate (IRC) calculations were performed to guarantee its corrected connection to the expected local minima. The final free energies were computed by summing the thermal corrections to Gibbs free energy with the single-point electronic energies. All quantum chemistry calculations were performed at the density

functional theory level as implemented in the Gaussian 16 program suite.

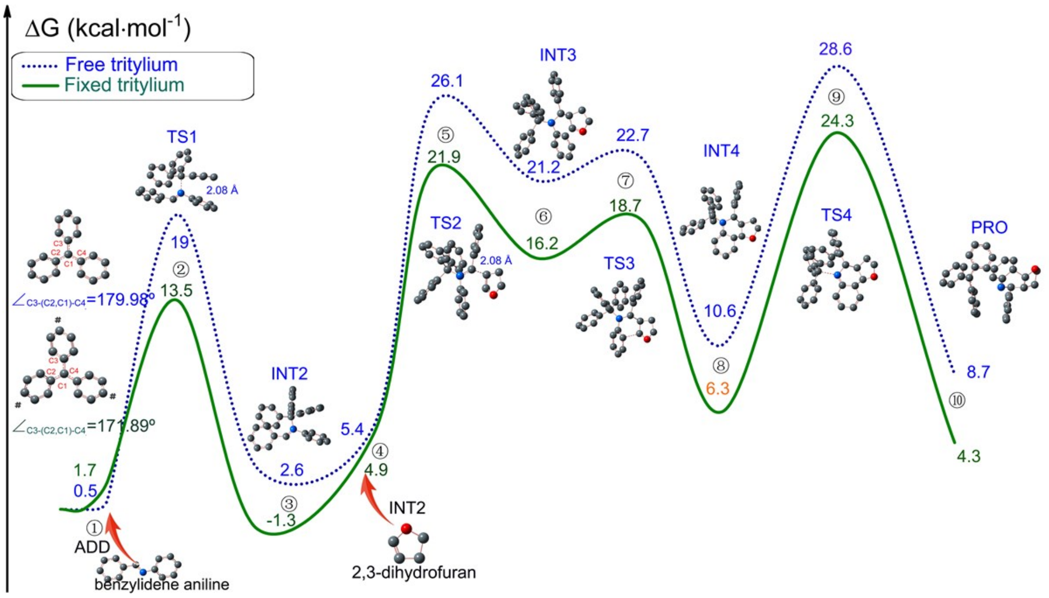

Fig. S12 Calculated free energy profile for the Povarov reaction of free (blue) and fixed (green) tritylium. Schematic representations of intermediates are inset. The fixed carbon atoms are marked by sign **#**. Hydrogen atoms are omitted for clarity. Gray C, blue N, red O.

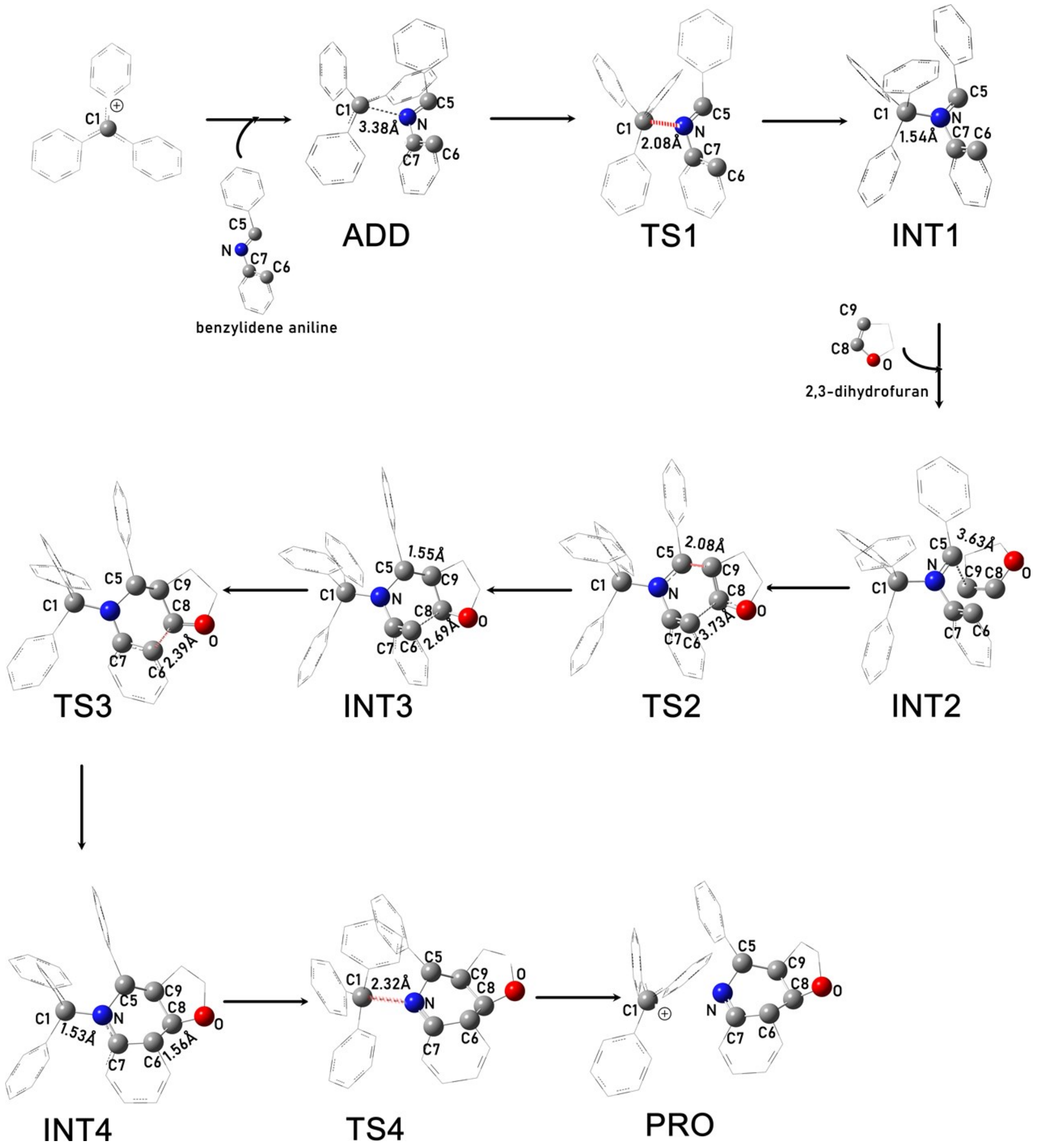

Fig. S13 Schematic diagrams of intermediates and transition species of a Povarov reaction using free or fixed tritylium catalyst and benzylidene aniline and 2,3-dihydrofuran as substrate. Hydrogen atoms are omitted for clarity. Gray C, blue N, red O.

11. ^{1}H NMR spectra of organic compounds

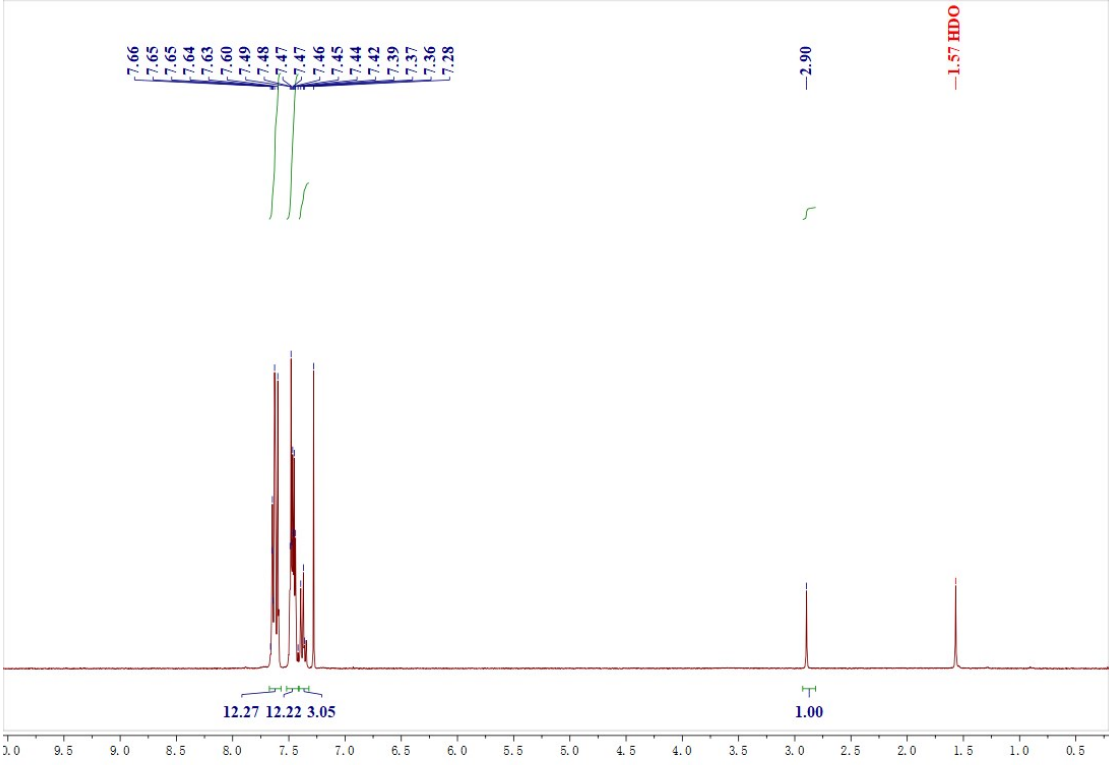

^{1}H NMR spectra of tri(4-biphenyl)carbinol (500 MHz, $CDCl_3$).

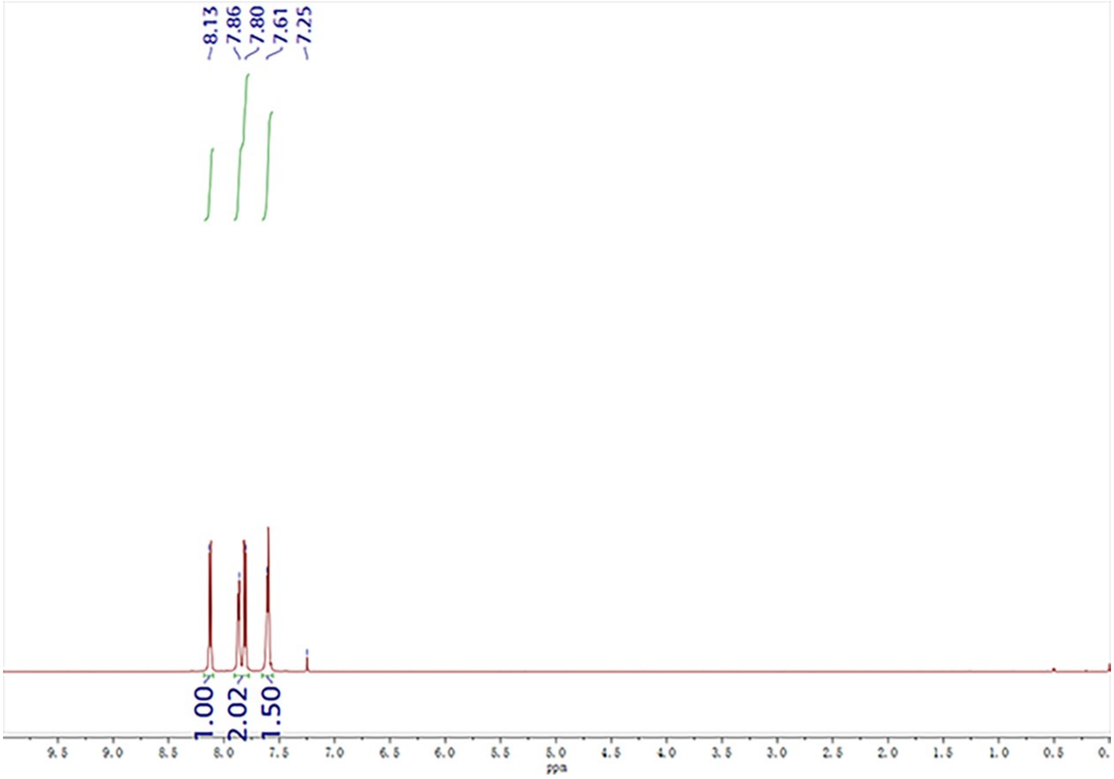

^{1}H NMR spectra of tri(4-biphenyl)carbonium tetrafluoroborate (500 MHz, $CDCl_3$).

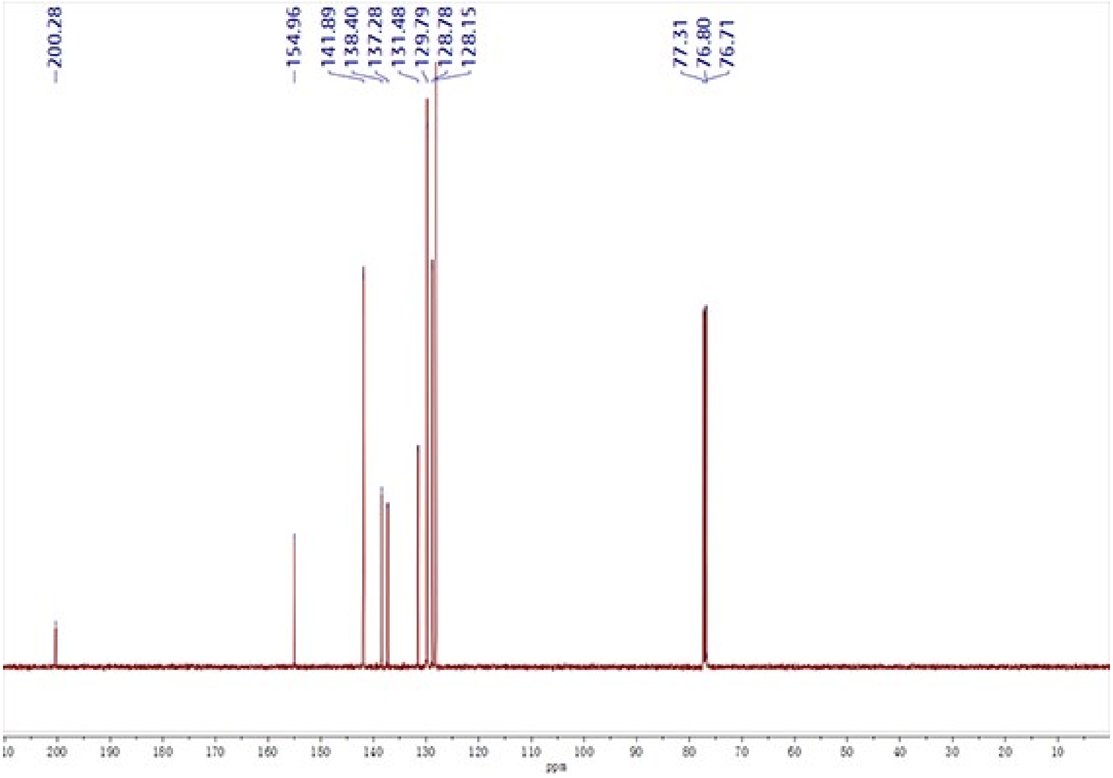

^{13}C NMR spectra of tri(4-biphenyl)carbonium tetrafluoroborate (125 MHz, $CDCl_3$).

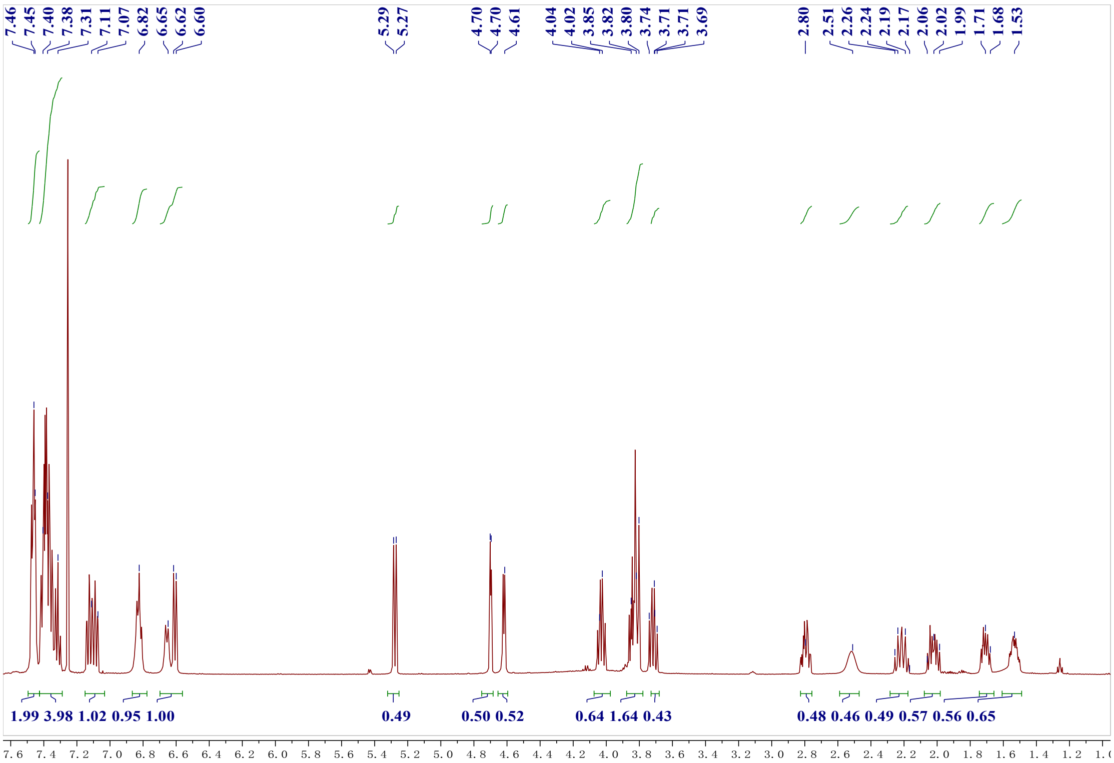

^{1}H NMR spectra of product **1** (Table 1, entry 2) (500 MHz, $CDCl_3$).

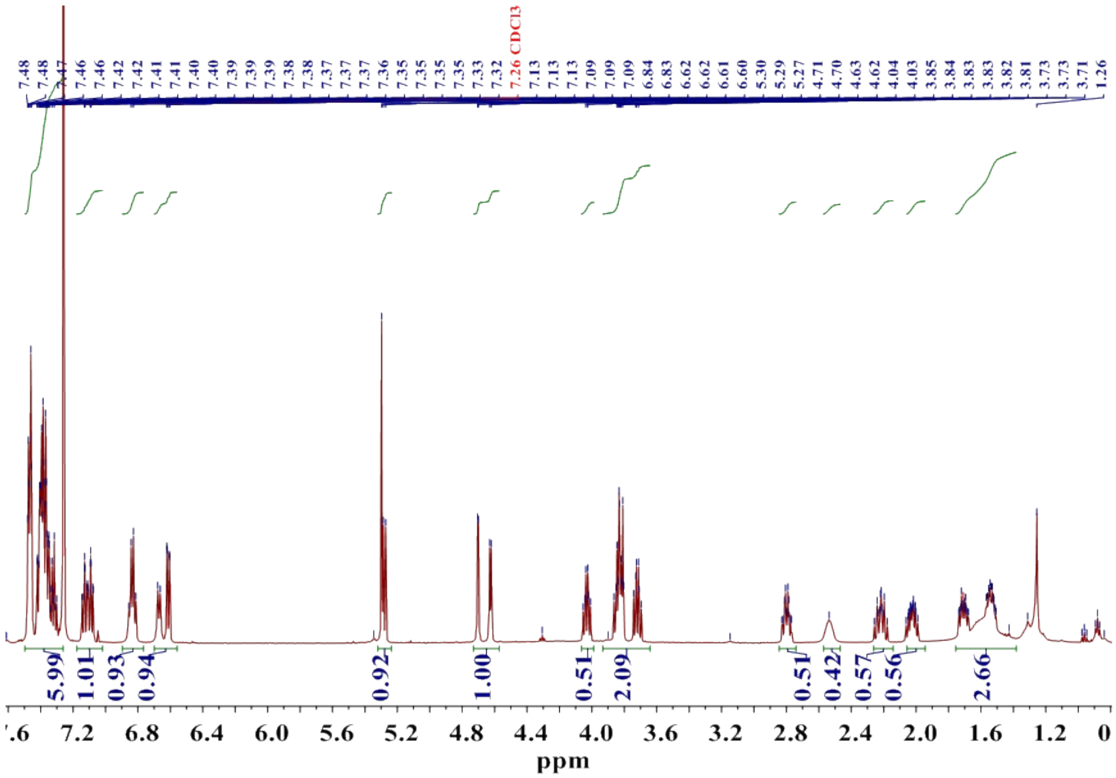

^{1}H NMR spectra of product **1** (Table 1, entry 5) (500 MHz, $CDCl_3$).

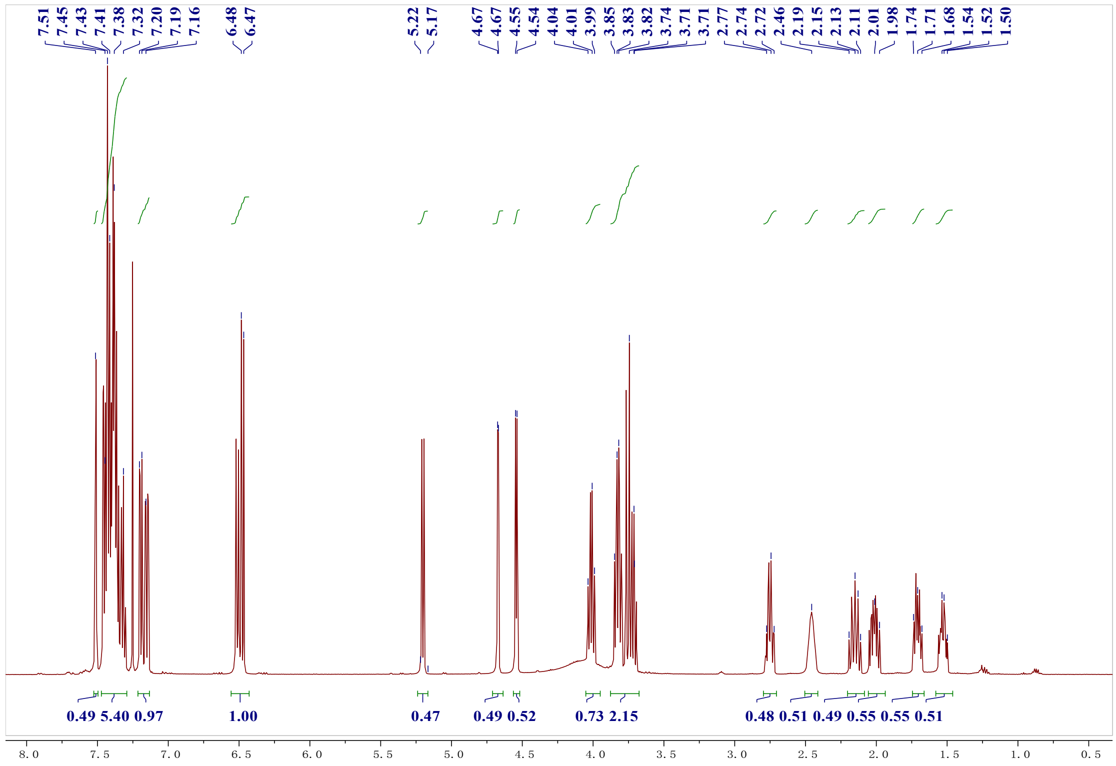

^{1}H NMR spectra of product **2** (Table 1, entry 7) (500 MHz, $CDCl_3$).

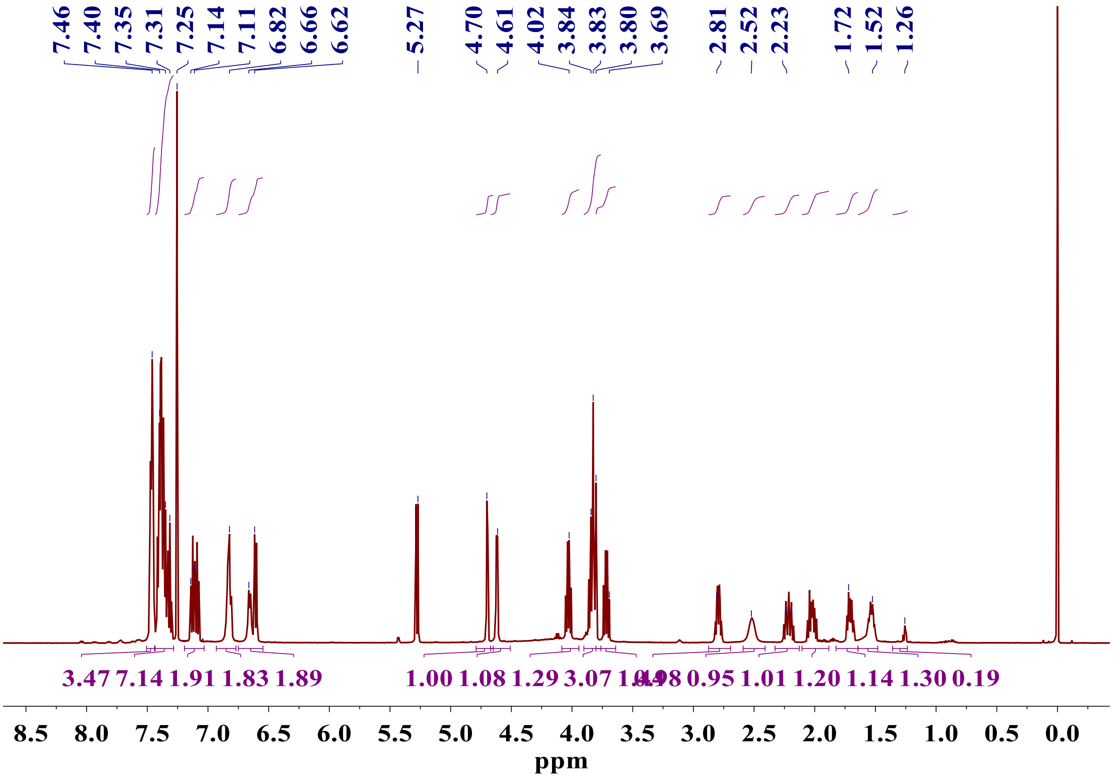

^{1}H NMR spectra of product **2** (Table 1, entry 8) (500 MHz, $CDCl_3$).

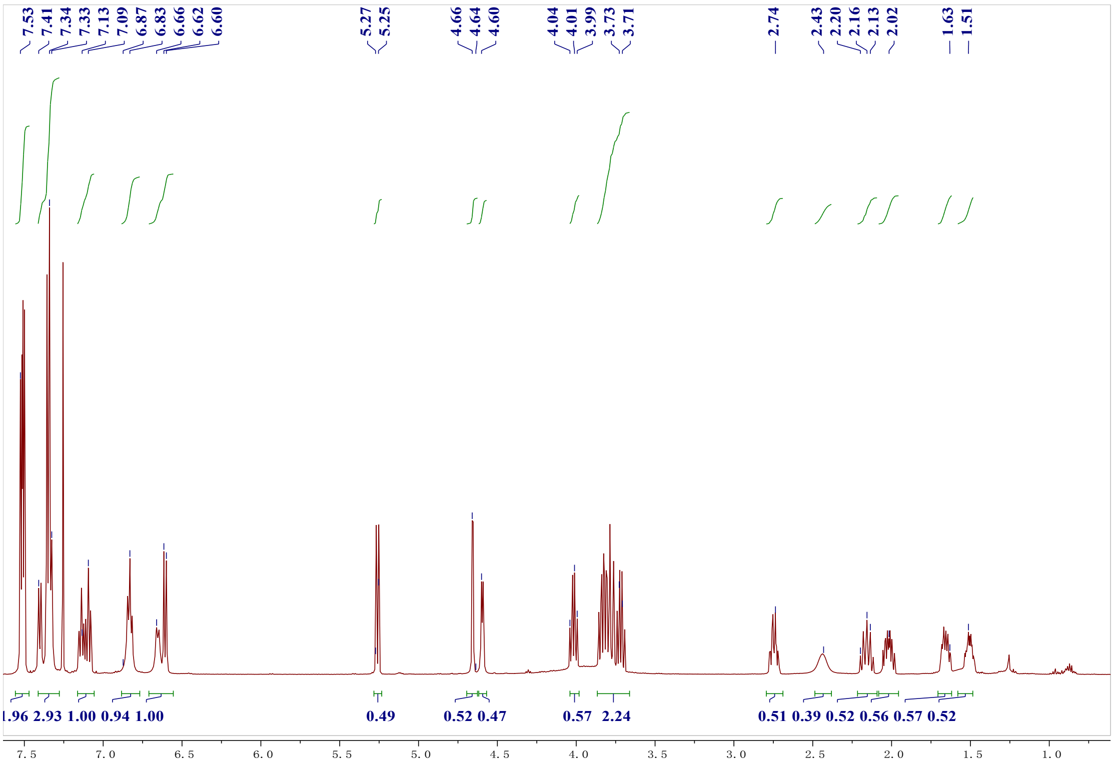

^{1}H NMR spectra of product **3** (Table 1, entry 9) (500 MHz, $CDCl_3$).

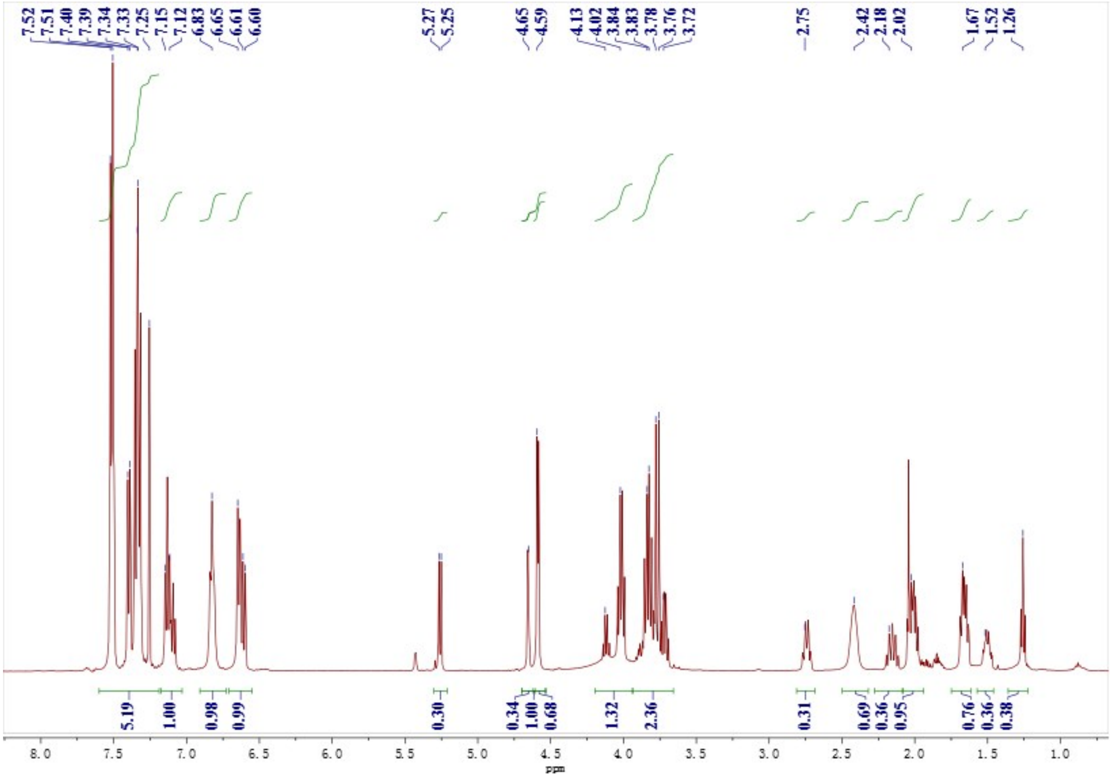

^{1}H NMR spectra of product **3** (Table 1, entry 10) (500 MHz, $CDCl_3$).

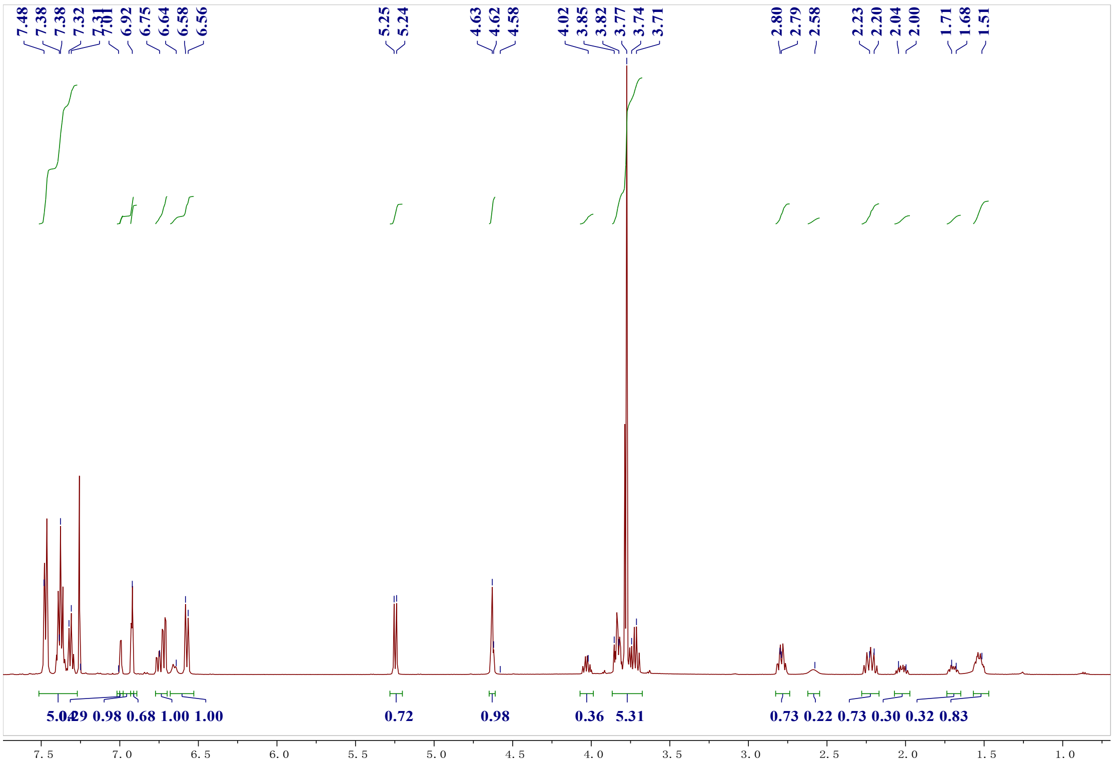

^{1}H NMR spectra of product **4** (Table 1, entry 11) (500 MHz, $CDCl_3$)

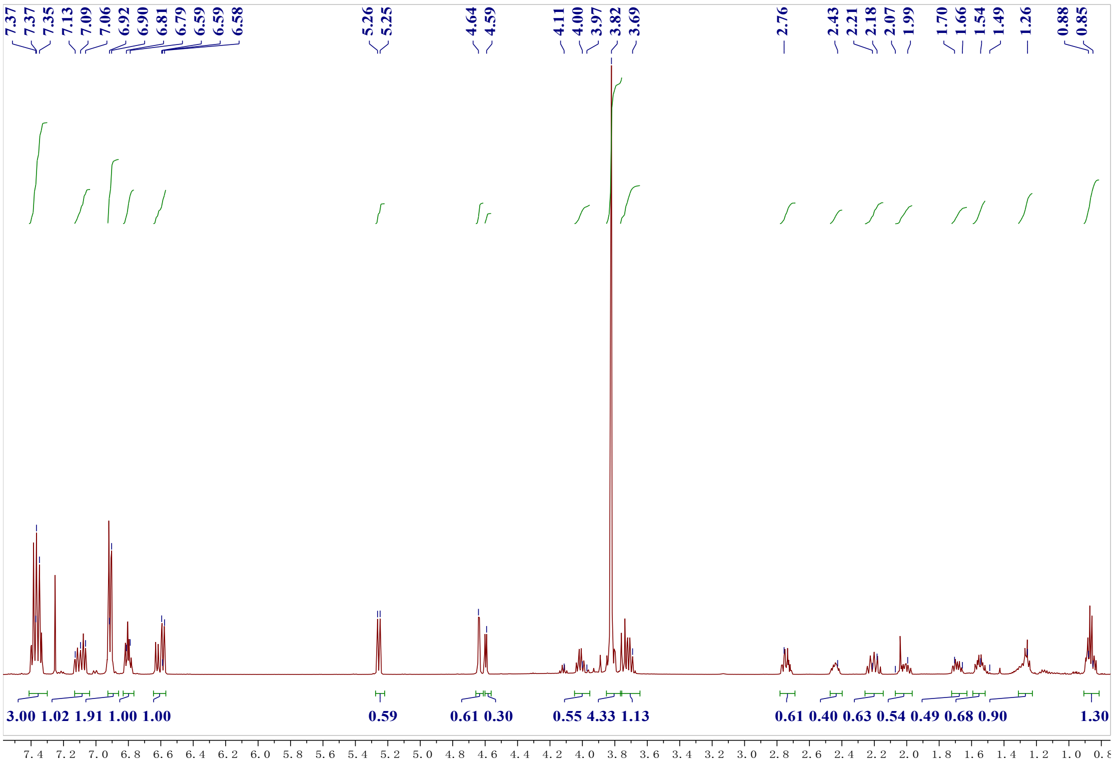

^{1}H NMR spectra of product **5** (Table 1, entry 13) (500 MHz, $CDCl_3$).

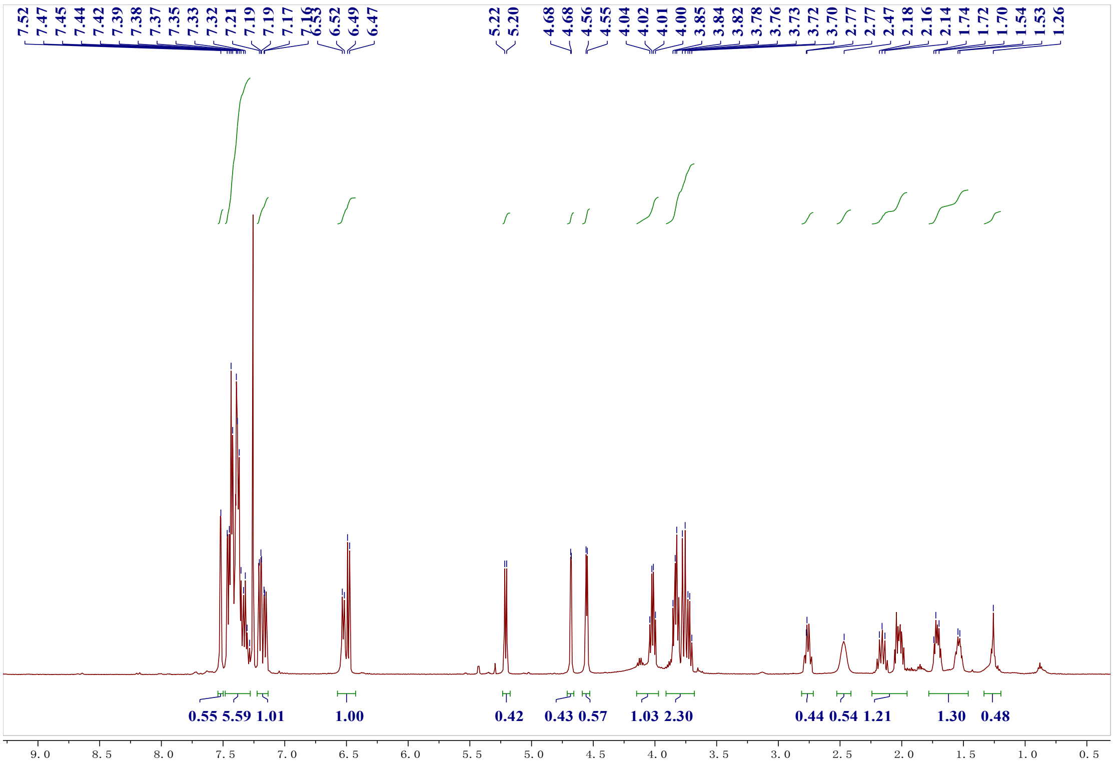

^{1}H NMR spectra of product **1** (Table 1, entry 15) (500 MHz, $CDCl_3$).

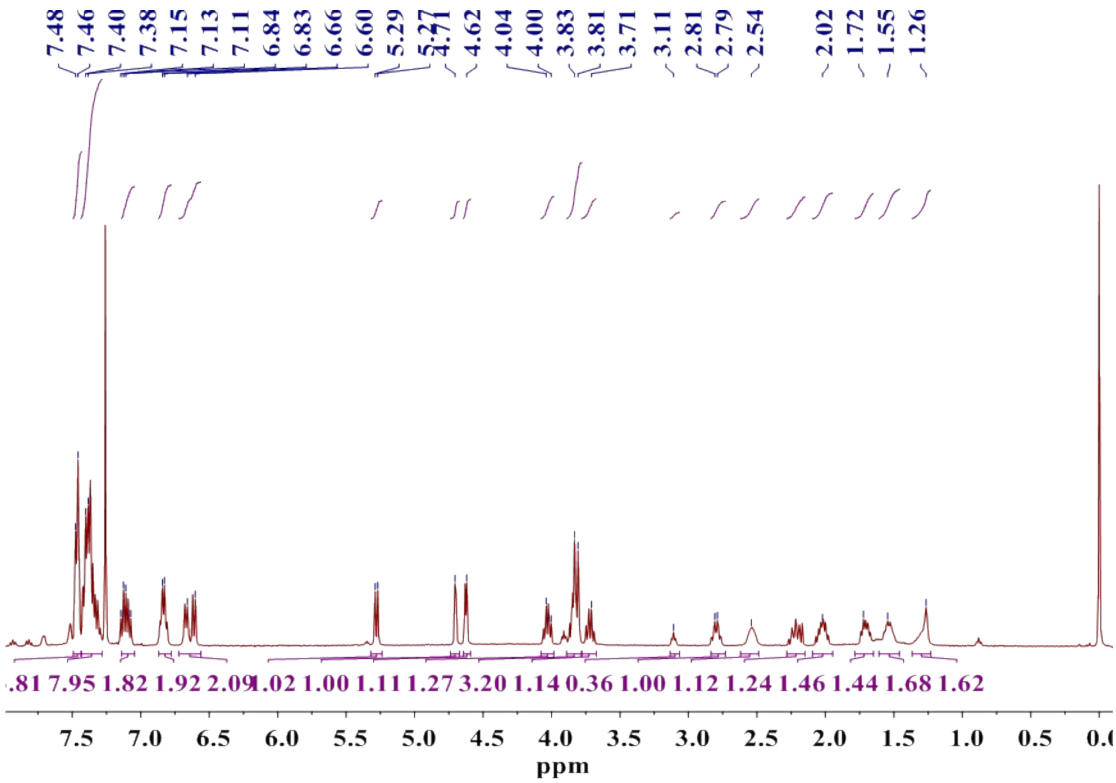

^{1}H NMR spectra of product **1** (Table 1, entry 16) (500 MHz, $CDCl_3$).

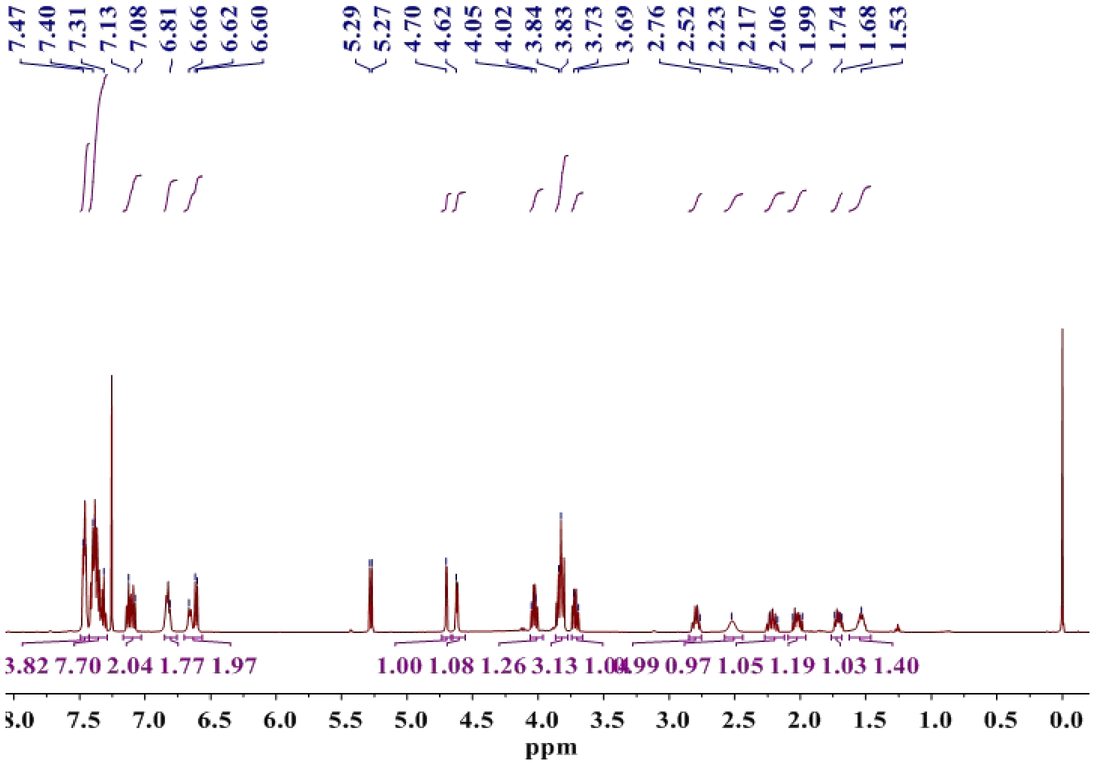

^{1}H NMR spectra of product **1** (Table 1, entry 17) (500 MHz, $CDCl_3$).

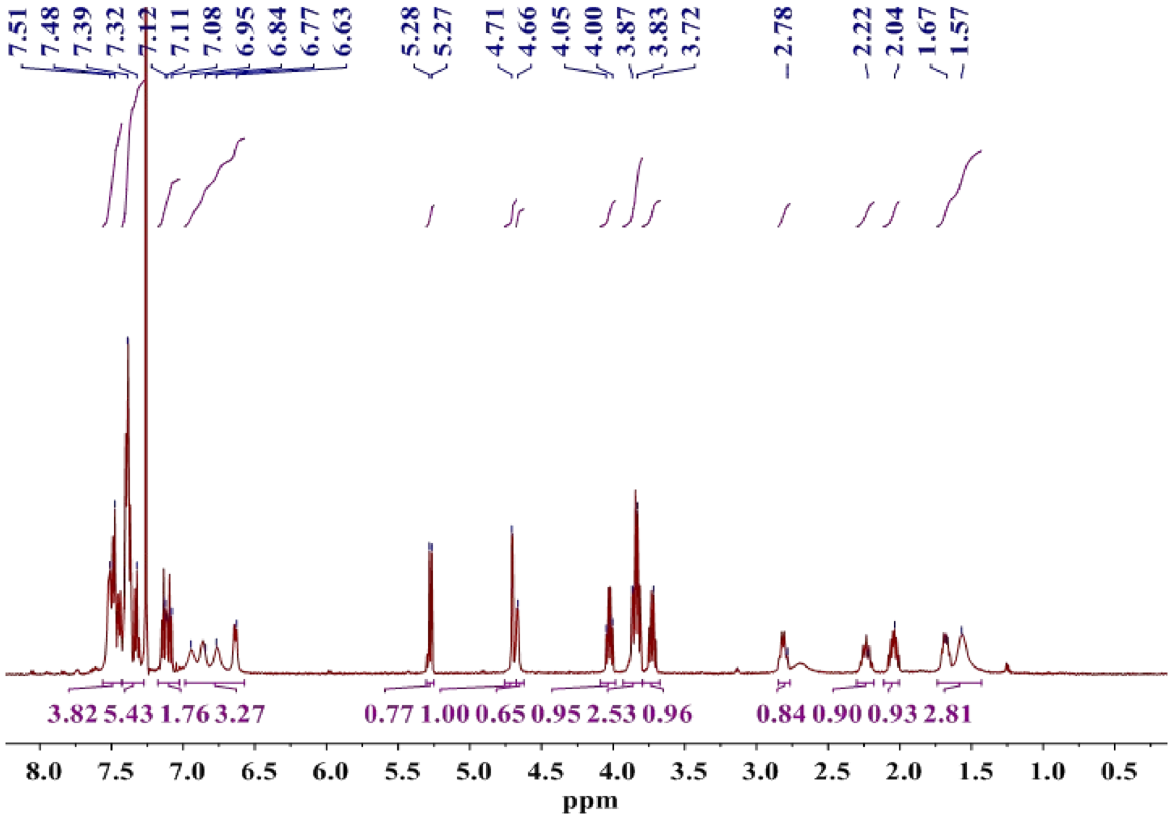

^{1}H NMR spectra of product **1** (Table 1, entry 18) (500 MHz, $CDCl_3$).

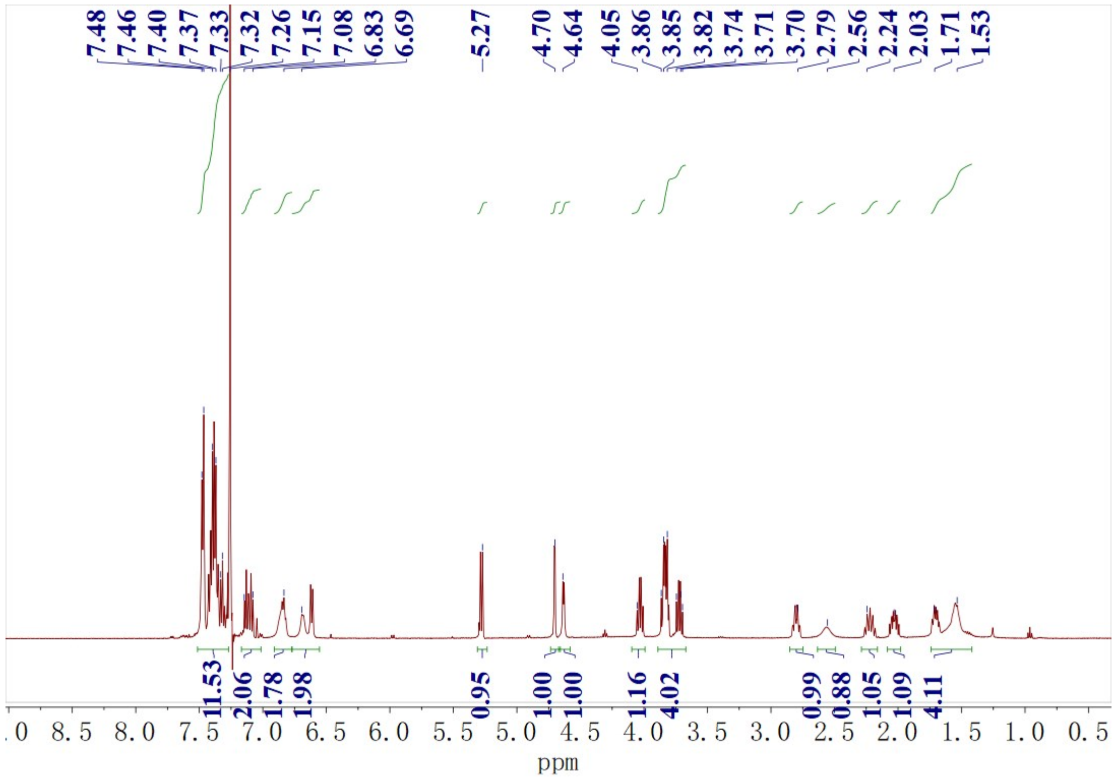

^{1}H NMR spectra of product **1** (Table 1, entry 19) (500 MHz, $CDCl_3$).

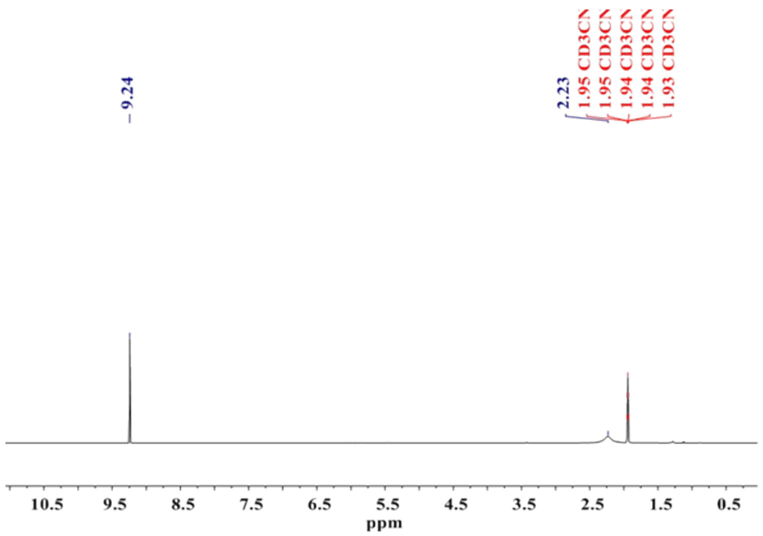

^1H NMR spectra of tropolium tetrafluoroborate (500 MHz, CD_3CN).

References

1 C. Wang, H. Yamamoto, *J. Am. Chem. Soc.*, 2014, **136**, 1222-1225.

2 T. Nishiuchi, S. Aibara and T. Kubo, *Angew.Chem. Int. Ed.,* 2018, **57**, 16516-16519.

3 J. Muñoz-Gomez, I. Marín-Montesinos, V. Lloveras, M. Pons, J. Vidal-Gancedo and J. Veciana, *Org. Lett.,* 2014, **16**, 5402-5405.
